# Supplementary material for: Membrane Fission Is Promoted by Insertion of Amphipathic Helices and Is Restricted by Crescent BAR Domains
Source: Cell. 2012 Mar 30;149(1):124–36. doi: 10.1016/j.cell.2012.01.047 (PMC3465558; doi:10.1016/j.cell.2012.01.047)
Supplement: Document S1. Article plus Supplemental Information [file mmc1.pdf]

Cell

Membrane Fission Is Promoted by Insertion of Amphipathic Helices and Is Restricted by Crescent BAR Domains

Emmanuel Boucrot,[1,3] Adi Pick,[2,3] Gamze Çamdere,[1,3] Nicole Liska,[1] Emma Evergren,[1] Harvey T. McMahon,[1,4,*] and Michael M. Kozlov[2,4,*]
[1]MRC Laboratory of Molecular Biology, Hills Road, Cambridge CB2 0QH, UK
[2]Department of Physiology and Pharmacology, Sackler Faculty of Medicine, Tel Aviv University, 69978 Tel Aviv, Israel
[3]These authors contributed equally to this work
[4]These authors contributed equally to this work
*Correspondence: hmm@mrc-lmb.cam.ac.uk (H.T.M.), michk@post.tau.ac.il (M.M.K.)
DOI 10.1016/j.cell.2012.01.047

SUMMARY

Shallow hydrophobic insertions and crescent-shaped BAR scaffolds promote membrane curvature. Here, we investigate membrane fission by shallow hydrophobic insertions quantitatively and mechanistically. We provide evidence that membrane insertion of the ENTH domain of epsin leads to liposome vesiculation, and that epsin is required for clathrin-coated vesicle budding in cells. We also show that BAR-domain scaffolds from endophilin, amphiphysin, GRAF, and β2-centaurin limit membrane fission driven by hydrophobic insertions. A quantitative assay for vesiculation reveals an antagonistic relationship between amphipathic helices and scaffolds of N-BAR domains in fission. The extent of vesiculation by these proteins and vesicle size depend on the number and length of amphipathic helices per BAR domain, in accord with theoretical considerations. This fission mechanism gives a new framework for understanding membrane scission in the absence of mechanoenzymes such as dynamin and suggests how Arf and Sar proteins work in vesicle scission.

INTRODUCTION

All eukaryotic cells rely on intracellular compartmentalization of vital processes within membrane organelles, whose shapes and dynamic interplay are tightly regulated to support their functions (Antonny, 2006; McMahon and Gallop, 2005; Shibata et al., 2009). Basic cellular compartments, including the endoplasmic reticulum (ER), the Golgi complex (GC), mitochondria, and intracellular transport intermediates (such as endocytic vesicles), contain in their structures highly curved tubular and spherical membrane elements undergoing persistent transformations and mutual conversion (McMahon and Gallop, 2005; Shibata et al., 2009). To form these intracellular membrane shapes, there are two essentially different types of membrane-sculpting events: generation of membrane curvature without disturbing membrane integrity and membrane remodeling by fission and fusion.

A lipid bilayer, constituting the structural basis of all cell membranes, resists both bending and remodeling (fission) (Chernomordik and Kozlov, 2003). Therefore, forces have to be applied and energy supplied to intracellular membranes in order to drive membrane curvature and fission. Several unrelated mechanisms have been suggested for protein-mediated membrane sculpting (Farsad and De Camilli, 2003; Antonny, 2006; McMahon and Gallop, 2005; Shibata et al., 2009) and scission (Chernomordik and Kozlov, 2003; Corda et al., 2006; Hurley and Hanson, 2010; Liu et al., 2009; Schmid and Frolov, 2011). The mechanisms of curvature generation by peripheral membrane proteins may be classified into two groups: (1) hydrophobic insertion mechanisms, based on penetration of hydrophobic or amphipathic protein domains into the lipid bilayer matrix, and (2) scaffolding mechanisms, where intrinsically curved and sufficiently rigid hydrophilic protein domains (or assemblies thereof) adhere to the lipid bilayer surface and impress their shapes on the membrane (McMahon and Gallop, 2005; Shibata et al., 2009). This has enabled a quantitative and unifying understanding of the action of practically all peripheral membrane proteins proven to date to generate membrane curvature. The state of the current understanding of membrane fission is less advanced. So far, several hypothetical models of membrane division have been suggested for Arf1 and dynamin (Beck et al., 2011; Roux et al., 2006; Schmid and Frolov, 2011) and for ESCRTIII (Hurley and Hanson, 2010), but these do not provide a quantitative basis on the forces driving membrane scission.

The present work establishes that shallow hydrophobic insertions, previously shown to generate membrane curvature, are sufficient to drive membrane fission resulting in the transformation of continuous membranes into separate vesicles. Previous work showed that the ENTH domain-containing protein epsin and N-BAR domain-containing proteins endophilin and amphiphysin could generate membrane vesicles in addition to the reported tubules with diameters from 20 to 50 nm (Ford et al., 2002; Gallop et al., 2006; Peter et al., 2004). This suggested

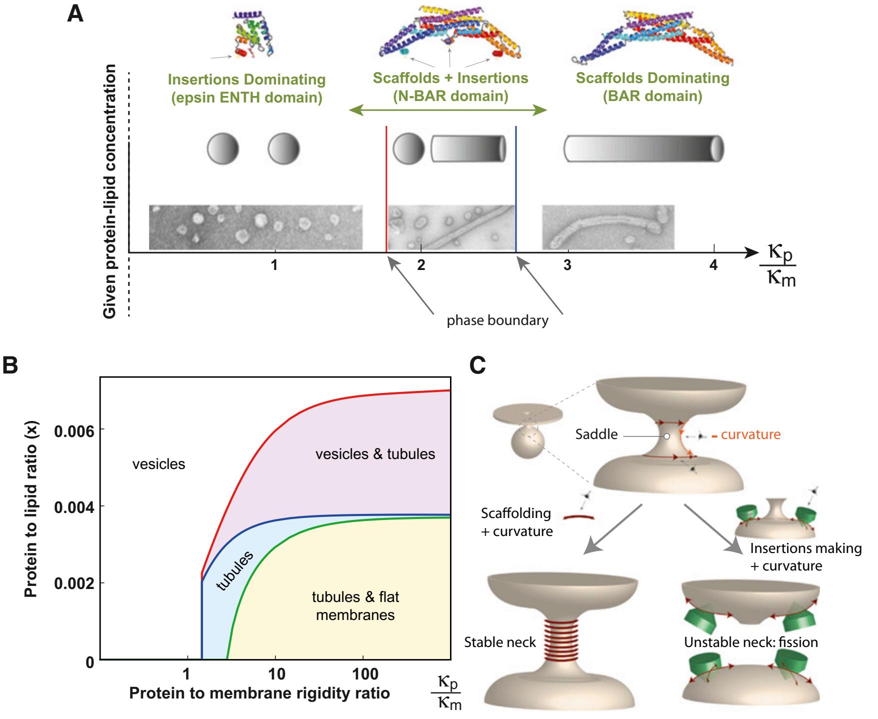

Figure 1. Predicted Membrane-Shaping Effect of Hydrophobic Insertions and Crescent-like Scaffolds

(A) Computationally predicted membrane configurations generated out of an initially continuous flat membrane by a combined action of hydrophobic insertions and crescent-like scaffolds. The predictions are based on model computations (see Extended Experimental Procedures), and presented as phase diagrams for different ratios between the bending rigidities of a protein scaffold and a lipid monolayer, κ_p/κ_m, characterizing the relationship between membrane shaping powers of insertions and scaffolds. Small values of κ_p/κ_m correspond to a prevailing effect of insertions, whereas large κ_p/κ_m values correspond to a strong effect of scaffolds. The parameters describing the insertions and scaffolds and the number of insertions per scaffold are taken as for amphiphysin N-BAR domain (see Extended Experimental Procedures). The specific values of κ_p/κ_m describing the transitions between different configurations correspond to those predicted for the amphiphysin-like N-BAR domains at the protein-to-lipid molar ratio x = 0.003 (see extended phase diagram in Figure S1D).

(B) Phase diagram for endophilin-like N-BAR module showing the ranges of the protein-to-lipid ratio, x, and the ratios κ_p/κ_m for which the initially flat membranes undergo bending and fission (vesicular state); bending without fission (tubular state), or coexistence of the two regimes (see Figure S1).

(C) Predicted effects of the hydrophobic insertions (green wedges) and crescent-like scaffolds (red scaffolds) on a saddle-shaped membrane neck connecting two membranes. A saddle has both positive (red) and negative (orange) curvatures. The scaffolds stabilize the neck into a tubule and, hence, prevent fission. The insertions destabilize the saddle-like shape of the neck, hence favoring fission.

that, in addition to promoting membrane curvature during endocytic vesicle formation (McMahon and Boucrot, 2011), ENTH and N-BAR domains could also promote membrane scission. As the common feature of these domains is the presence of membrane-inserting amphipathic helices at their N termini, we hypothesize that this structural module might be the key factor necessary and, likely, sufficient for membrane fission.

A theoretical analysis was conducted of the elastic energy of small vesicles and membrane tubules, using a coarse-grained model, accounting effectively for the molecular features of lipids and proteins. This analysis predicted that proteins containing shallow insertion domains promote membrane scission, whereas a protein whose membrane interaction face is crescent-like, such as crescent BAR domains (without insertions or twists), which bend membranes by the scaffolding mechanism, prevent membrane fission, hence, counteracting membrane insertions. We validated these predictions using a new in vitro quantitative vesiculation assay and found a crucial role for epsin during clathrin-coated vesicle (CCV) budding in cells.

RESULTS

Predictions from a Biophysical Model

Membrane fission involves rearrangements of membrane continuity requiring specialized protein modules. To foresee the effect of shallow hydrophobic insertions and/or crescent-like protein scaffolds on membrane fission, we undertook a comparative analysis of system energies in tubular and vesicular states based on a coarse-grained semiquantitative physical model (see Extended Experimental Procedures). The results can be presented in the form of phase diagrams (Figures 1A and 1B; Figure S1 available online) predicting formation of the vesicular state, tubular state, and coexistence between them for different protein-to-lipid ratios, x, and different ratios between the scaffold and lipid bilayer bending moduli, κ_p/κ_m. The latter parameter characterizes the ability of scaffolds to generate membrane curvature. Vanishing values of this parameter, $\kappa_p/\kappa_m = 0$, describe proteins that do not produce any scaffolding effect and bend membranes solely by shallow insertion of amphipathic helices. The larger κ_p/κ_m, the stronger the scaffolding effect. Qualitatively an increase of κ_p/κ_m is equivalent to a decrease in the number of amphipathic helices per scaffold for a given protein rigidity κ_p (Figure 1A). The extended phase diagram in Figure 1B corresponds to an N-BAR with an extra amphipathic domain in the middle (such as endophilin). The points corresponding to $\kappa_p/\kappa_m = 0$ describe scaffold-less proteins such as epsin ENTH domains. Complete modeling is presented in the Extended Experimental Procedures.

The major conclusion illustrated by the phase diagrams (Figures 1A, 1B, and S1D–S1F) is that shallow hydrophobic insertions are predicted to be sufficient for vesicle formation, driving membrane fission, whereas crescent-like protein scaffolds are predicted to support formation of continuous membrane tubules, hence disfavoring fission (Figures 1A, 1B,

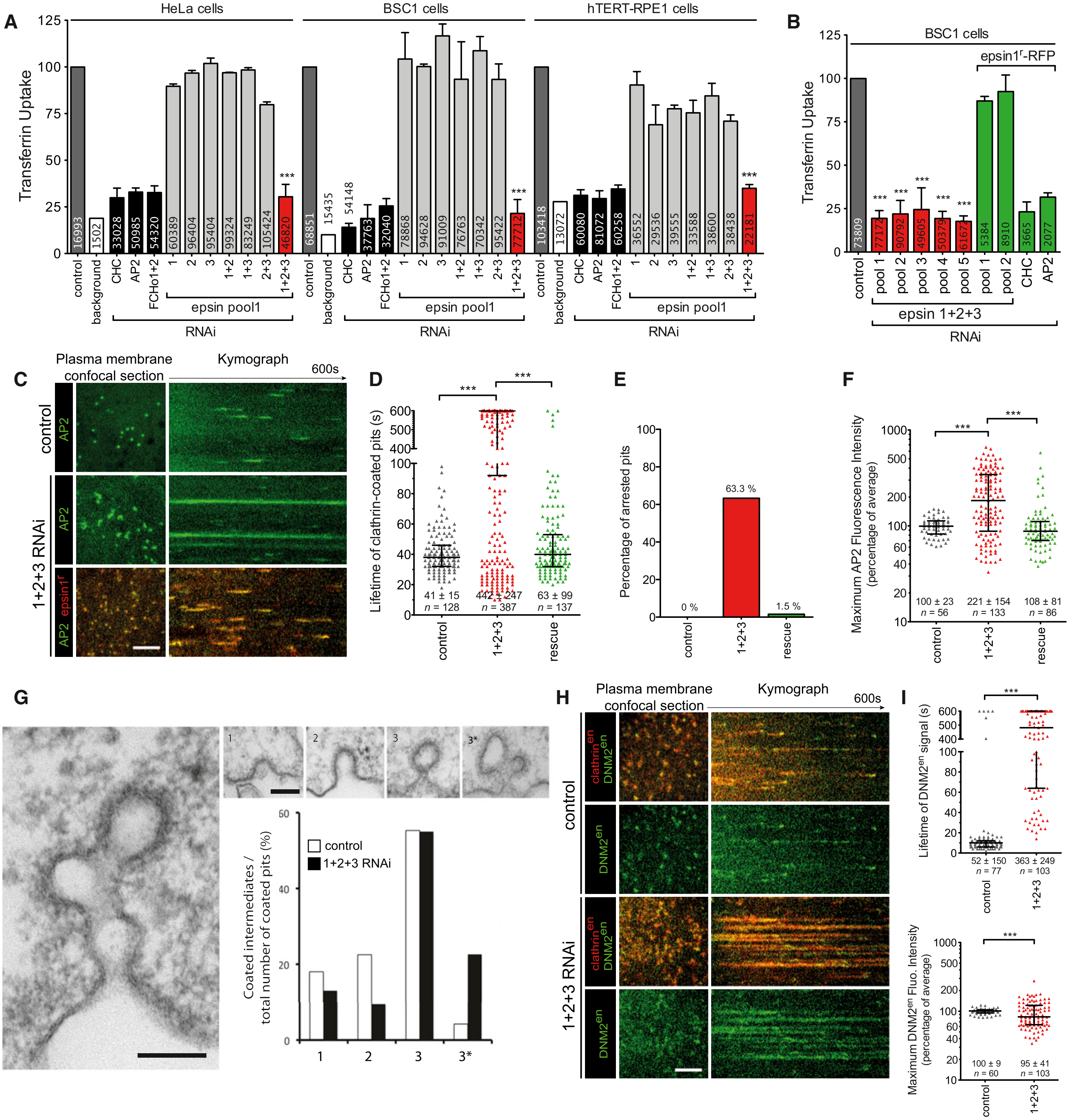

Figure 2. Epsin Is Required for CCV Scission

(A) Effect of RNAi (siRNA pool1) of epsin proteins on transferrin (Tf) uptake measured by flow cytometry. Clathrin (CHC), AP2, and FCHo proteins depletion were used as positive controls (black bars). The values were normalized to the mean of the control cells (gray bars). The background (cells without Tf) for each cell line is shown (white bars). The number of cells analyzed is displayed on each bar. ***p < 0.0001. Data are the mean ± SD.

(B) Effects of 5 independent pools of siRNA against Epsin1+2+3 (red bars) on Tf uptake and of the rescue of pools 1 and 2 (but not CHC and AP2 RNAi) by coexpression of rat epsin1-RFP (green bars). Experiments were done as in (A). Data are the mean ± SD.

(C) Effect of epsin1+2+3 RNAi on the dynamics of clathrin-coated structures (CCS) and rescue by coexpression of rat epsin1-RFP. CCS labeled by σ2-EGFP. Bar, 5 μm.

(D) Scatter plots of individual lifetimes of CCS from three different cells, measured on data set similar to (C). Median with interquartile range is shown on graph and mean ± SD is written at the bottom, *n* is the number of events analyzed. ***p < 0.0001.

(E) Fraction of CCS with longer duration than the time series.

and S1). The mechanistic background for these predictions comes from a qualitative consideration of the mechanical stability of a funnel-like membrane neck, an unavoidable intermediate stage of the fission process (Figure 1C). Fission occurs if this neck is unstable, i.e., possesses extra energy, which can be released as a result of membrane scission. Geometrical considerations show that the saddle-like shape of the neck membrane is characterized by having a larger midplane area than outer and inner leaflet surface areas (occupied by lipid head groups). Expansion of the head group region with respect to the bilayer midplane would stress and destabilize the neck, hence, favoring its fission. This reasoning can be expressed in exact terms of the insertion contribution to the membrane modulus of Gaussian curvature and the role of the latter in determining membrane conformations (Huse and Leibler, 1991; Schwarz and Gompper, 1999).

Shallow hydrophobic insertions, such as amphipathic helices from proteins like epsin, span mainly the polar head regions of membrane monolayers and do not penetrate deeply into the monolayer hydrocarbon region (Kweon et al., 2006). As a result, these insertions expand the bilayer surface(s) with respect to the bilayer midplane and, hence, are predicted to destabilize the neck and favor membrane fission. Although this effect is strongest if insertions are introduced into both membrane monolayers (expanding the head groups region on both sides), estimations show that amphipathic helices inserted only in the outer monolayer at biologically reasonable concentrations can be sufficient to drive fission on their own. Moreover, insertions are predicted not only to make fission energetically favorable but also to accelerate this reaction by reducing its energy barrier. The fission rate was previously proposed to be limited by the energy of the membrane stalk intermediate (Bashkirov et al., 2008; Kozlovsky and Kozlov, 2003), and computations show that a positive contribution to outer monolayer spontaneous curvature generated by the insertions (Campelo et al., 2008) decreases the stalk energy (Kozlovsky and Kozlov, 2003). Taken together, shallow hydrophobic insertions are expected to support membrane fission into small vesicles both in terms of the overall energy balance and kinetically.

Crescent-like scaffolds, such as BAR and F-BAR domains that do not penetrate lipid monolayers, do not change the area balance between membrane surfaces and the midplane. Instead, they mold, locally, the membrane into a cylindrical shape, which is curved only along the line of the scaffold-membrane interface (Figure S1H). The energetically most favorable situation for multiple crescent-like scaffolds is where they are oriented parallel to each other on a tubular surface. Hence, pure crescent-like scaffolds are not expected to support membrane fission but rather are predicted to generate tubular shapes (Figure S1).

"Hybrid" proteins, such as N-BAR domains, with both insertion and scaffolding effects, are predicted to generate coexisting vesicles and tubules with the degree of preference for the former or latter depending on the amount of the amphipathic helices per scaffold and on the effective rigidity of the scaffold, which includes the strength of the scaffold binding to the membrane surface (Figures 1A, 1B, and S1). Hence, scaffolding by BAR domains is predicted to restrain membrane fission mediated by hydrophobic insertions, and BAR domains with an increasing number of amphipathic helices are predicted to support increasing membrane vesiculation (Figures S1D–S1I) and so potentially in vivo will be on the pathway to membrane fission.

Epsin Is Required for CCV Scission

Epsin proteins were initially chosen as a paradigm for insertion activity in the absence of scaffolding. Epsin proteins play a role in cargo selection and membrane sculpting of CCV (Ford et al., 2002; Wang et al., 2011) but have not been linked so far with membrane fission. The lack of endocytic defects in epsin1 and 2 double knockout-derived cells (Chen et al., 2009) and in cells depleted of epsin1 by RNA interference (RNAi) (Chen and Zhuang, 2008; Kazazic et al., 2009) is likely due to protein redundancy, as there are at least four epsin proteins in humans: epsin1, -2, and -3 and epsinR (Clint/enthoprotein). EpsinR is involved in CCV formation from intracellular compartments (Mills et al., 2003), whereas the remaining epsins are believed to function from the plasma membrane. We measured the effects of individual or combinatorial depletion of epsin1, -2, and -3 by RNAi on clathrin-mediated endocytic activity as measured by transferrin (Tf) uptake (Figure 2A). We found that only simultaneous depletion of epsin1, -2, and -3 (1+2+3 RNAi) led to a significant decrease in Tf uptake, giving a similar effect to depletion of clathrin, FCHo proteins (Henne et al., 2010), or AP2 (Figures 2A, S2A, and S2B). This phenotype was specific as it was confirmed using up to five different 1+2+3 siRNA pools (comprising 24 different siRNAs) in three different cell lines and could be specifically rescued by coexpression of rat epsin1-RFP, which was resistant to the epsin1 siRNA in pools 1 and 2 (Figures 2B and S2A). Rat epsin1-RFP did not rescue, as expected, clathrin or AP2 RNAi. The perturbation was specific to clathrin-mediated endocytosis, as the uptake of the fluid-phase marker dextran was not affected (Figure S2C). Because epsin3 is known to be upregulated in some tumors (Coon et al., 2011), the phenotype was confirmed in a normal diploid cell line (hTERT-RPE1) where we have shown by mass spectrometry that epsins 1, 2, and 3 are all expressed (Figures 2A and S2B). The block of Tf uptake upon codepletion of epsins was largely due to a defect in scission of clathrin-coated structures (CCS) as epsin 1+2+3 RNAi cells had most (63.3%) of their AP2- and clathrin punctae arrested

(F) Scatter plots of individual maximum fluorescence intensities of CCS from three different cells. Data are presented as in (D), excepted for the Log10 vertical axis. ***$p < 0.0001$.

(G) Morphological analysis of CCS in BSC1 cells treated or not with epsin1+2+3 RNAi. Representative electron microscopy images for various categories quantified (top). Bars, 100 nm. Coated structures were classified as 1, shallow; 2, invaginated; 3, constricted; and 3*, multiheaded. Repartition between the various categories of 70 structures from control (white bars) and 1+2+3 RNAi (black bars) cells is shown. Large image on left and 3* image are from RNAi-treated cells.

(H) Effect of epsin1+2+3 RNAi on recruitment of endogenous dynamin 2 ($DNM2^{en}$, green) and clathrin ($CLTA^{en}$, red). Bar, 5 μm.

(I) Scatter plots of individual lifetimes (top) and individual maximum fluorescence intensities (bottom) of endogenous dynamin2. Data are presented as in (D) and (F), respectively.

See also Figure S2.

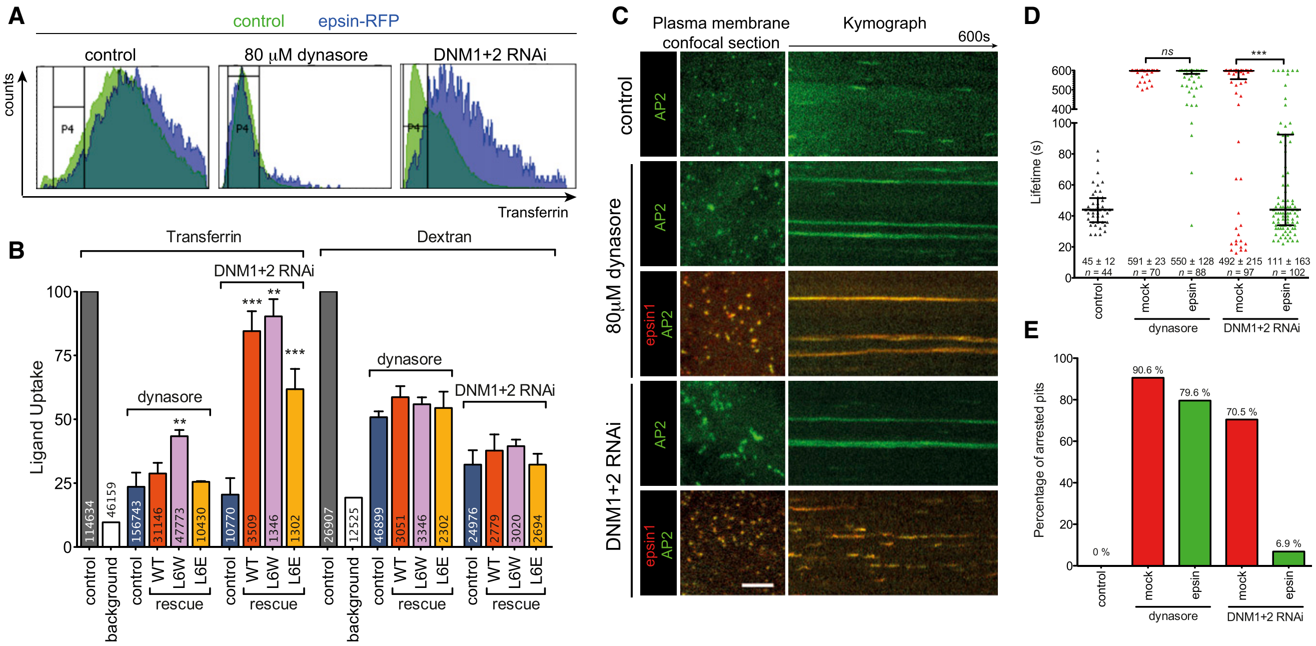

Figure 3. Epsin Can Mediate CCV Scission in Dynamin-Depleted Cells

(A) Representative FACS profiles of transferrin (Tf) uptake in BSC1 cells treated with 80 μM dynasore or dynamin (DNM) 1+2 RNAi as in (B) and with (blue) or without (green) rat epsin1-RFP expression.

(B) Effect of rat epsin1-RFP (wt), L6W and L6E mutants on Tf uptake or dextran uptake in cells treated with 80 μM dynasore or DNM1+2 RNAi. The values were normalized to the mean of the control cells (gray bars). The background (cells without Tf or dextran) is shown (white bars). Number of cells analyzed is displayed on each bar. ***p < 0.0001, **p < 0.001. Data are the mean ± SD.

(C) Effect of rat epsin1-RFP coexpression on CCS (labeled by σ2-EGFP) dynamics in cells treated with 80 μM dynasore or DNM1+2 RNAi as in (B). Bar, 5 μm.

(D) Scatter plots of individual lifetimes of CCS from three different cells, measured on data sets similar to (C). Median with interquartile range is shown on graph and mean ± SD is written at the bottom, *n* is the number of events analyzed. *ns*, nonsignificant; ***p < 0.0001.

(E) Fraction of CCS with longer duration than the time series.

See also Figure S3.

and enlarged ("1+2+3," Figures 2C–2F). These defects were rescued by the re-expression of rat epsin1-RFP ("rescue," Figures 2C–2F). By electron microscopy (EM), the number of CCS per μm cell perimeter was not significantly different in the control and epsin 1+2+3 RNAi cells (control: 0.065 ± 0.042 CCS/μm, n = 70; 1+2+3 RNAi: 0.070 ± 0.036 CCS/μm, n = 70; p > 0.05). The relative abundance of different stages of CCS—shallow, invaginated, and constricted—were similar in the two samples. However, a marked increase in the number of multiheaded CCS were observed in the epsin 1+2+3 RNAi sample, representing 23% of the total number of pits versus 4% in the control (Figure 2G). Large patches of flat clathrin-coated plasma membrane were also observed, reminiscent of what Brodin and colleagues (Jakobsson et al., 2008) observed when interactions of epsin with clathrin and AP2 were perturbed acutely in the giant lamprey synapse. The diameter of CCVs still attached to the membrane was not significantly different in RNAi-treated cells (p > 0.05, Student's t test; control 106 nm, 1+2+3 RNAi 102 nm; n = 50), but the neck diameter of constricted coated pits (stage 3) was significantly greater (p > 0.001, Student's t test; control 25.7 nm, 1+2+3 RNAi 35.0 nm; n = 50). The defect in scission in epsin 1+2+3 RNAi cells was not due to a lack of recruitment of dynamin as both dynamin 1 and 2 were detected for significantly longer times and at higher intensities at the arrested CCS (Figures 2H, 2I, and S2E), although from live cell imaging we do not know if it is present on the neck. Altogether, these studies reveal that epsin is required for CCV scission.

Epsin Can Mediate CCV Scission in Dynamin-Depleted Cells

To test whether epsin could support CCV budding independently of dynamin, we tested the potential rescue by slight overexpression of epsin in two situations where dynamin function was impaired: when dynamin was locked at the neck (using the small-molecule dynamin inhibitor dynasore) and when dynamin was depleted (dynamin1 and 2 [DNM1+2] RNAi). Mild overexpression of epsin did not significantly rescue the CCV budding defect induced by dynasore, but did rescue DNM1+2 RNAi, as judged by Tf uptake (Figures 3A and 3B) and the rescue in clathrin-AP2 dynamics (Figures 3C and 3D). This suggested that epsin can support the scission of the neck of CCS when dynamin expression is reduced (DNM1+2 RNAi) but not when dynamin is locked at the neck (dynasore). The ability of epsin to promote CCV scission with dynamin RNAi was sensitive to its amphipathic helix insertion (Figures 3B and S3). Mutation of a charged residue on the hydrophobic face of the amphipathic helix L6E (reducing membrane binding and curvature induction

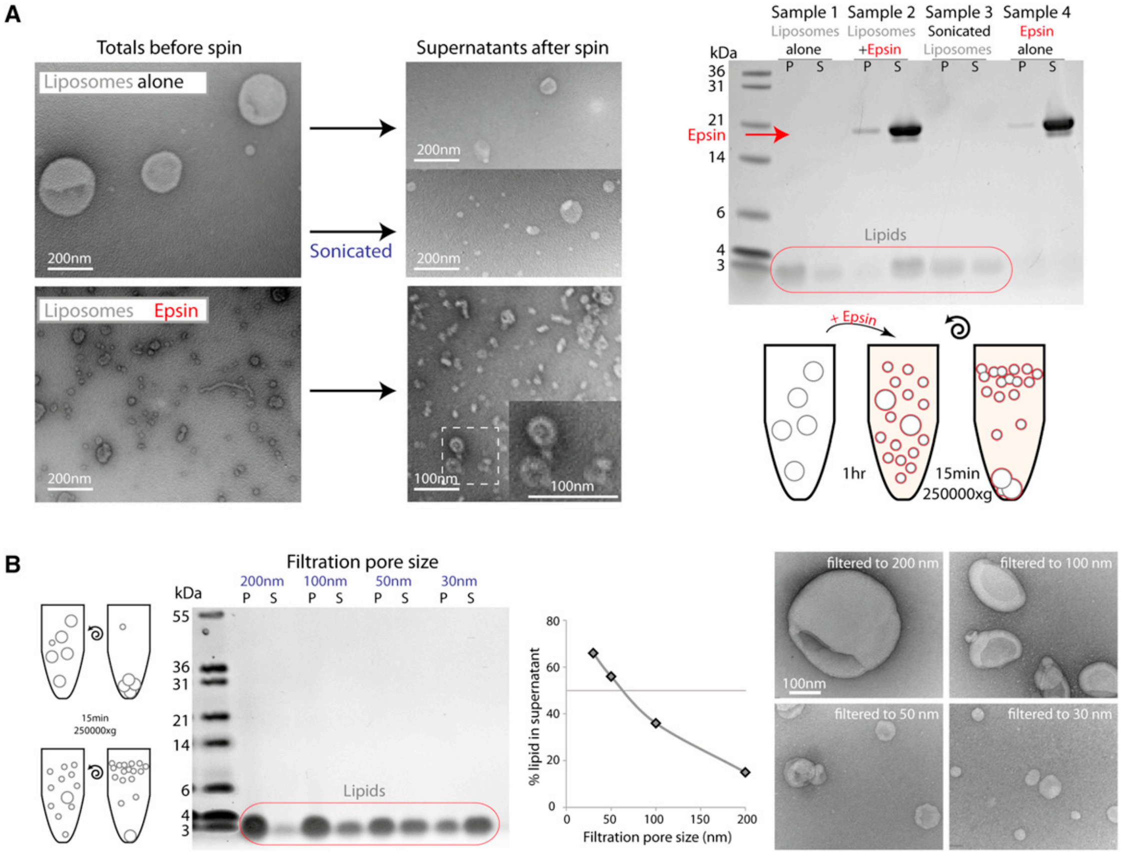

Figure 4. Epsin ENTH Domain Causes Extensive Membrane Vesiculation

(A) Epsin (10 μM) incubated with 0.125 mg/ml Folch liposomes for 1 hr at room temperature. Samples for electron microscopy were taken before and after centrifugation. For sonicated liposomes a sample was subjected to probe sonication for 5 s. Samples for centrifugation were spun as indicated in the diagram. Pellets (P) were resuspended in the same volume of buffer as the supernatant (S). Lipids and proteins were visualized as described in Experimental Procedures.
(B) Folch liposomes filtered to various sizes were subjected to centrifugation and the lipid distribution was assessed by SDS-PAGE. Samples for electron microscopy were taken before the spin. A more complete distribution of vesicle sizes is shown in Figure S4C.
See also Figure S4.

[Ford et al., 2002]) did not rescue scission to the same extent (despite being recruited to CCS) as wild-type (WT) epsin or a mutant with increased membrane binding, L6W (Ford et al., 2002). Thus, we concluded that epsin supports CCV scission and works alongside dynamin.

Epsin ENTH Domain Causes Membrane Vesiculation

The membrane-binding face of epsin ENTH domain has an intense positively charged patch (Figure S4A), which allows the domain to be recruited to negatively charged membranes, where it binds to PtdIns(4,5)P_2 (PIP_2), inducing the folding of an N-terminal sequence into an amphipathic helix forming a pocket for the head group of this lipid (Ford et al., 2002). The folding of this helix is relatively specific for PIP_2, where three residues of the helix are involved in hydrogen bonding with the lipid. This exposes a hydrophobic surface, which along with surrounding hydrophobic residues is proposed to sit in the hydrophobic phase of the membrane (Figures S4A and S4B).

When incubated with liposomes, epsin ENTH domain forms many small nanovesicles and extremely narrow tubules of ~20 nm diameter (Figure 4A). An assay was needed to quantify the nanovesicle formation. In a standard lipid cosedimentation assay, proteins that bind to liposomes generally pellet (P) with the liposomes whereas soluble proteins that do not bind remain in the supernatant (S). When we performed this assay with epsin ENTH domain, it was puzzling that the protein appeared to remain in the supernatant even in the presence of PIP_2-containing Folch liposomes (Figure 4A, Samples 2 and 4). This indicated either that the protein did not bind or that the membranes were now in the supernatant fraction. To monitor the membrane distribution after velocity sedimentation, we exploited our observation that Coomassie dye stains both proteins and lipids on the same SDS-PAGE gel (where stain and fix have no alcohol so as not to dissolve the lipids). Liposomes (filtered to 200 nm) were found in the pellet fraction as visualized by Coomassie staining of the lipids close to the dye front of the gel (Figure 4A, Sample 1). However, on addition of epsin ENTH domain to liposomes, the lipid signal moved to the supernatant fraction (Figure 4A, Sample 2). Thus the protein must have interacted with the liposomes and changed the apparent density. A partial shift

could also be achieved by sonication of the liposomes (Figure 4A, Sample 3). This observation forms the basis of an unbiased biochemical assay for membrane vesiculation, where small vesicles were found to resist pelleting. EM of the starting material compared to liposomes in the presence of epsin showed dramatic membrane vesiculation of the starting material and possible micelle formation (Figure 4A, lower EM panel). The increased number of nanovesicles in the presence of epsin was consistent with the vesiculation of 200 nm liposomes to 20 nm giving at least a 100-fold increase in vesicle number. After sedimentation only small liposomes were found in the supernatants of any of these samples (Figure 4A).

To assess the dynamic range of the assay, we filtered liposomes using polycarbonate membrane filters with defined pore sizes. Liposomes filtered to a diameter of 200 nm sedimented efficiently, whereas liposomes filtered to 30 nm did not (Figure 4B). Electron microscopy confirmed that the liposomes were indeed filtered to approximately the defined size (with some heterogeneity) and that vesicles with diameters smaller than 100 nm tended to resist pelleting. (The broad transition between flotation and pelleting also partially reflects the range of diameters achieved with the filtration process; Figure S4C). It is likely that more highly curved liposomes have a greater contribution from lipids to their apparent density (on centrifugation) than larger liposomes, leading to a difference in pelleting, consistent with previous observations (Goormaghtigh and Scarborough, 1986). We concluded that the relative distribution of lipids between pellet and supernatant in these experiments is an unbiased biochemical measure of the extent of vesiculation and that this bulk assay agrees with the EM observation of extensive vesiculation of liposomes by epsin ENTH domain.

Vesiculation of liposomes by the epsin ENTH domain was concentration dependent with maximal vesiculation around 2.5 μM protein (Figure S4D, but see comment later). If all protein was bound to the membrane surface and an individual epsin occupies an area equivalent to 20 lipids (Figure S4B) then at 0.125 mg/ml lipid the membrane would be 70% saturated. As can be seen in the saturation curve, vesiculation occurred at much lower concentrations but did not go to completion in the time given (Figure S4D).

To test whether the observed flotation of small vesicles is limited to the particular Folch extract mix used in these experiments, we made a synthetic mixture containing 10% cholesterol, 5% PIP_2, 55% PC, and 30% PS (used to achieve a strong electrostatic attraction for epsin, as would be expected in the plasma membrane inner leaflet where the protein binds in vivo). The addition of epsin resulted in robust vesiculation as determined by the sedimentation assay (Figure S4E).

Epsin-Mediated Vesiculation Is due to Amphipathic Helix Insertion

To understand the nature of epsin-dependent vesiculation, we next tested epsin mutants. Epsin L6W resulted in a slight increase in vesiculation compared to WT after 1 hr at 37°C (Figure 5A). Samples taken for electron microscopy after 5 min incubations showed that L6W resulted in uniform small vesicle production, whereas WT protein gave tubules and vesicles with a wide distribution of sizes (Figures 5A–5C). Experiments at 4°C showed a large increase in vesiculation with L6W over WT protein (Figure S5A). After 1 hr incubation, vesiculation by L6E was less efficient than WT ENTH domain at 37°C (Figure 5A), and no vesiculation was observed either at 4°C (Figure S5A) or in the 5 min time point processed for electron microscopy (Figure 5B). Vesicles produced by L6W for 5 min at 37°C had diameters centered around 20 ± 4 nm, whereas WT protein had a much broader distribution (Figures 5C and S5B). This would indicate that epsin works in a stochastic manner to bud vesicles off larger structures, and that stable tubule intermediates are not required on the way to vesiculation (as predicted by our theoretical model).

Altered vesiculation that accompanies mutations of epsin's amphipathic helix points to the importance of this module in vesicle generation. However, vesiculation may not be a direct property of the helix but simply a reflection of the amount of protein bound to membrane, where the helix can be considered as an anchor. This is particularly plausible because at 4°C we observed that only L6W, which binds membranes much better than WT protein, led to vesiculation (Figure S5A). An alternative strategy to determine the importance of the amphipathic helix was to exploit the PIP_2 dependence of helix folding (Ford et al., 2002). One PIP_2 binds per one epsin molecule, and thus if one epsin covers approximately 20 lipids (Figure S4B) then 5% PIP_2 should allow complete saturation of the membrane. With 10 μM epsin ENTH domain, there was complete vesiculation of this lipid mix (Figure 5D). As expected, this resulted in a dramatic shift of lipids from the pellet to the supernatant after a high-speed spin, which correlated with vesiculation as judged by electron microscopy. To reduce the amphipathic helix concentration in the membrane 10-fold, we lowered the PIP_2 content to 0.5%. This also resulted in maximal vesiculation (Figure 5D). As a control, there was no vesiculation when PIP_2 was removed altogether, despite significant association of the protein by electrostatic attraction to the PS-containing membranes. Thus, our experiments show that a relatively low density of amphipathic helices (1 helix to 200 lipids) is required to achieve maximal vesiculation (as determined by movement of membranes into the supernatant in the sedimentation assay). We do not observe epsin dimer formation on membranes and the low concentration of protein required for vesiculation argues against a molecular crowding model for membrane vesiculation. From these measurements, we can calculate that formation of a 20 nm vesicle with 10% coverage of the membrane by epsin ENTH footprints will require at least 30 molecules. Calculations based on the spontaneous curvature of epsin (Campelo et al., 2008) and bilayer curvature of a 20 nm vesicle show that about 100 molecules are required (in very close agreement with the biochemical measurement).

To further address effects of epsin ENTH domain amphipathic helix insertion into membranes, we tested for trypsin sensitivity of this sequence, which has multiple lysine and arginine residues and is proposed to be unfolded in solution (Ford et al., 2002). Limited proteolysis gave a distinct cleavage product of 1–2 kDa and a corresponding decrease in molecular mass of the parent protein (Figure 5E). By mass spectrometry, we identified the cleaved peptide as a fragment of the amphipathic helix (Figure S5C). This cleavage was protected by liposomes, showing that it is inserted (unlike a soluble synaptobrevin

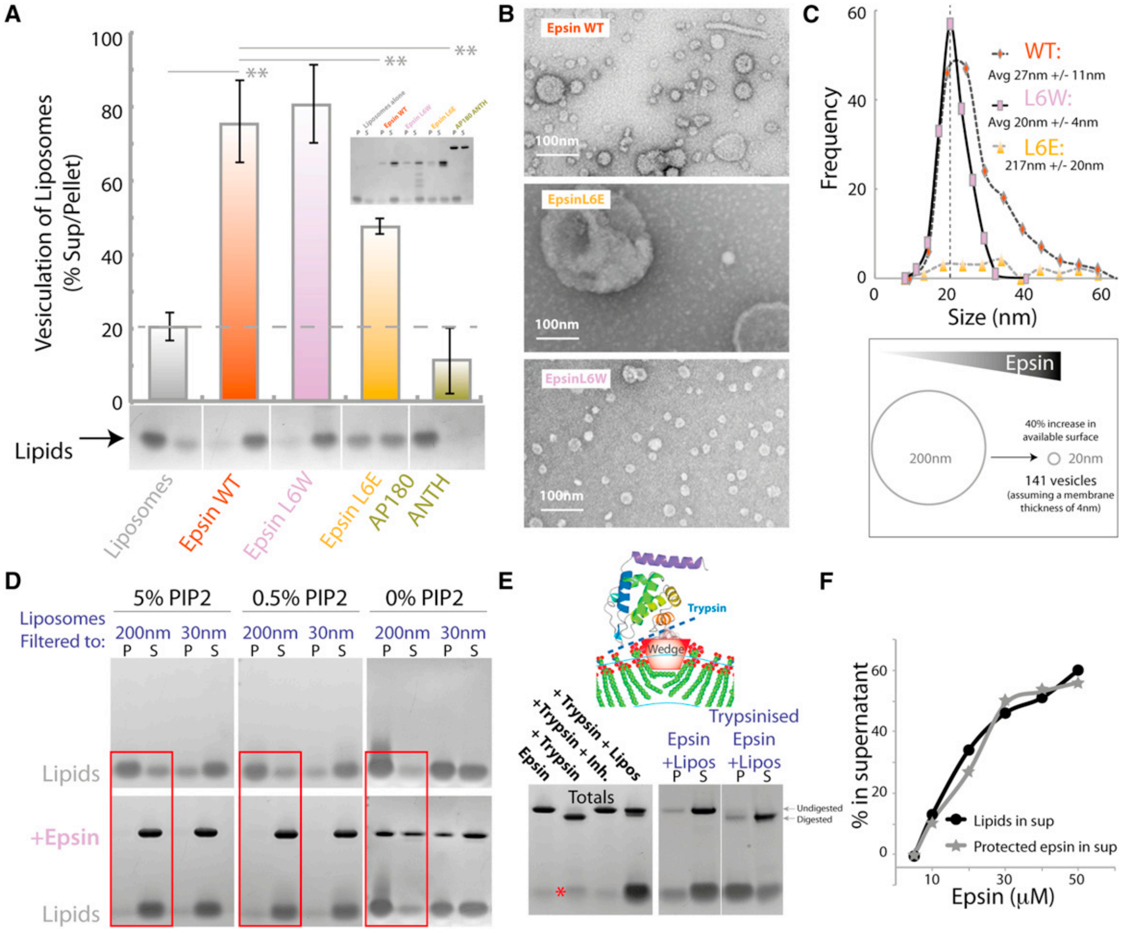

Figure 5. Membrane Vesiculation Is due to Amphipathic Helix Insertion

(A) Membrane vesiculation due to epsin ENTH domain and mutants was assessed by the biochemical vesiculation assay and by electron microscopy. Protein (10 μM) was incubated with 0.125 mg/ml Folch + 5% PIP_2 liposomes for 1 hr at 37°C. AP180 ANTH domain, which also binds to PIP_2 (Ford et al., 2001), was used as a control. Data are mean of three experiments ± SD with a sample gel shown on the right. **$p < 0.001$.

(B) Electron microscopy samples taken for samples in (A) after 5 min.

(C) Quantitation of membrane vesiculation after 5 min incubation with WT and L6W epsin ENTH domain. The WT protein gives a broader distribution of vesicle sizes with many vesicles of larger diameters. Data in each case are from 169 objects in at least three different fields. One 200 nm vesicle is estimated to give 141 vesicles of 20 nm.

(D) PIP_2 dependence of epsin vesiculation. Epsin ENTH (10 μM) was incubated for 1 hr with either 200 nm or 30 nm-filtered synthetic liposomes (30% PS, 10% cholesterol, 55%–60% PC plus indicated amount of PIP_2, final concentration of liposomes: 0.125 mg/ml). We see no effect of protein addition on the 30 nm-filtered liposomes. Vesiculation is dependent on PIP_2, but binding can still be observed.

(E) Limited trypsin proteolysis (20 min at 37°C) of epsin ENTH domain was inhibited by Soybean trypsin inhibitor (Inh.) or by liposomes (left). *For cleaved peptide sequence, see Figure S4C. The amphipathic helix was either pretrypsinized or not before addition of liposomes (right).

(F) The amount of vesiculation shows a strong correlation with the amount of epsin protected, as assessed by a trypsin assay in (E). Thus it is not so important to know the amount of epsin added or membrane bound but the amount of helix insertion.

See also Figure S5.

fragment, Figure S5D). The stability of epsin for up to 30 min in the presence of trypsin+membranes showed that epsin did not dissociate at a significant rate. Cleaved ENTH domain no longer bound at a significant level to membranes (Figure 5E). As a proof of principle of helix insertion, we showed that reduced PIP_2 levels led to reduced helix protection/insertion and with 0% PIP_2 there was no protection (Figure S5E). We could also show that there was a strong correlation between the protection of epsin from trypsin cleavage and vesiculation (Figures 5F and S5F). The assay also allowed us to look at membrane "binding/insertion" of our different epsin mutants where we could show that the L6W mutant bound more tightly than WT protein (Figure S5G).

Positive Correlation of the Number of Hydrophobic Insertions and Membrane Fission by N-BAR-Domain Proteins

To examine the prediction that BAR domains restrain amphipathic helix-induced membrane fission, we tested the effects of various BAR and N-BAR proteins on liposome morphology. Endophilin A1 (EndoA1) has previously been observed to give

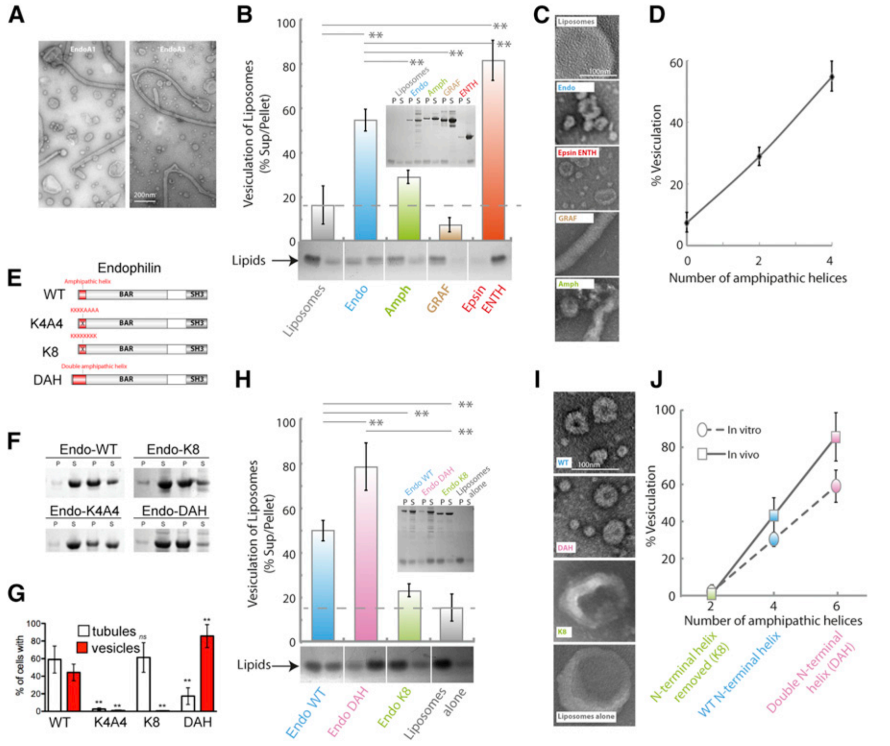

Figure 6. BAR Scaffolds Restrain Membrane Scission Catalyzed by Extensive Hydrophobic Insertions

(A) Membrane tubulation and vesiculation by endophilins A1 and A3. Full-length proteins (4 μM) were incubated with liposomes at 0.5 mg/ml for 15 min at room temperature and then prepared for EM.

(B) Vesiculation by Endo (full-length EndoA3), Amph (full-length amphiphysin 2–6), GRAF (GRAF1 BAR+PH domain), and Epsin (epsin1 ENTH domain). Folch liposomes at 0.125 mg/ml were incubated for 1 hr at 37°C. EndoA3 (2 μM), 4 μM Amph, and 8 μM GRAF was used (increasing concentrations were used to compensate for potentially reduced binding with less hydrophobic insertions). Data are the mean of three experiments ± SD. A sample gel is included. **p < 0.001.

(C) Corresponding EMs of samples in (B) before sedimentation. Larger areas of the grids are shown in Figure S3.

(D) Correlation between the extent of hydrophobic insertions and vesiculation.

(E) Schematic representation of the endophilin mutants used. EndophilinA3 WT has an N-terminal amphipathic helix (red), a BAR domain (BAR), and a C-terminal SH3 domain (SH3). Endo-DAH has a Double N-terminal Amphipathic Helix. Endo-K4A4 and Endo-K8 have, respectively, four lysines (K4) and four alanines (4A) or eight lysines (K8), instead of their N-terminal amphipathic helices. Experiments were conducted with untagged proteins.

(F) Membrane binding for WT and helix mutants. Protein (4 μM) was incubated for 15 min at room temperature with excess Folch liposomes to avoid vesiculation. Liposomes were added to the right two lanes in each panel.

(G) Histogram showing the percentage of transfected cells displaying internal tubules (white) and internal vesicles (red). Cells could present both. Data are the mean ± SD of >300 cells for each construct from three independent experiments. ns, nonsignificant, **p < 0.001.

(H) Vesiculation by Endo-WT DAH and K8. Liposomes (0.125 mg/ml) were incubated for 1 hr at 37°C with 2 μM protein. Data are the mean of three experiments ±SD. A sample gel is included. **p < 0.001.

(I) EMs of samples taken before the sedimentation in (H). Larger areas of the grids are shown in Figure S6.

(J) Graph showing the extent of vesiculation with different numbers of amphipathic helices. Data taken from (H) and (J).

See also Figure S6.

a mixture of vesicles and tubules formed from larger liposomes (Gallop et al., 2006; Figure 6A). Here we used endophilin A3, a form of the protein expressed in nonneuronal tissue and localizing to membranes in fibroblasts (Hughes et al., 2004), thus allowing us to also test the phenotypes in vivo (see below). EndoA1 and EndoA3 generated a mixture of vesicles and tubules (Figures 6A and S6) from 200 nm liposomes. In the biochemical vesiculation assay almost 60% of the starting material was vesiculated in 1 hr (Figure 6B). By comparison, amphiphysin2 (Amph) had higher than background vesiculation but was significantly less active than EndoA3. GRAF, which does not have any amphipathic helices (Lundmark et al., 2008), was inactive in vesiculation (Figure 6B). Epsin ENTH domain showed over 80% vesiculation in the same incubation. EM observation of the samples confirmed these results (Figures 6C and S6A). The degree of vesiculation correlated strongly with the number of amphipathic helices. Thus endophilin with four amphipathic helices (N terminus and middle of the BAR domain) had higher activity compared to amphiphysin which only has two N-terminal amphipathic helices, which was better than GRAF with no amphipathic helices (Figure 6D).

Given that vesicles were observed for epsin, endophilin, and amphiphysin, we wondered what effect the BAR domains have on the final product of vesiculation. We thus measured the size distribution of vesicles generated by the various BAR proteins. We already noted above that epsin makes 20 nm vesicles (outer diameter). Endophilin vesicles have a wider distribution with an endophilin mutant showing an average diameter of 27 nm (Figure S6J), whereas amphiphysin vesicles are around 47 nm (Figure S6B). Vesicles of diameter 30 and 45 nm were previously observed for EndoA1 and Amph N-BAR domains, respectively (Peter et al., 2004; Gallop et al., 2006) (Figure S6C). Thus, an increased number of amphipathic helices on a BAR domain correlates with increased vesiculation and smaller vesicle size, as predicted by the quantitative assessment based on membrane physics.

Scaffolding by BAR Domains Restrains Membrane Fission

To test the balance between scaffolding and hydrophobic insertion, we altered the hydrophobicity of the N-terminal amphipathic helix of endophilin A3 (Endo-WT). To shift the

membrane bending capacity toward predominant scaffolding, we replaced the N-terminal amphipathic helix with stretches of four or eight lysines (K4A4 and K8, Figure 6E) to compensate for the reduced membrane binding in the absence of the N-terminal amphipathic helix (Gallop et al., 2006). To shift the protein toward the other extreme of a more pronounced hydrophobic insertion, we doubled the N-terminal amphipathic helix (double amphipathic helix [DAH]). Membrane binding of purified proteins showed that Endo-K8 had similar binding to Endo-WT (Figure 6F). In contrast, Endo-K4A4 bound less well and Endo-DAH bound membranes slightly better than the WT protein (Figure 6F). All four endophilin constructs were recruited as expected to plasma membrane punctae in cells (Figure S6D), suggesting proper folding and functionality. The majority of cells expressing Endo-WT had many internal tubules and/or vesicles labeled with protein (Figures 6G and S6D). Individual tubules were very dynamic and often vesiculated during observations (Figures S6E–S6G). In contrast, cells expressing Endo-DAH had more internal vesicles (85% ± 13%) and less tubules (17% ± 10%) than cells expressing Endo-WT, with most of these tubules being very short (Figures 6G and S6D). Virtually all (91% ± 2%) Endo-DAH tubules observed vesiculated (Figure S6F). Compared to Endo-WT, Endo-DAH vesiculated sooner after formation (Figure S6G). These Endo-positive intracellular punctae were indeed endocytic membrane vesicles, as they labeled positive after a pulse with FM4-64 (Figure S6H). The majority of the cells expressing Endo-K8 had tubules (Figures 6G and S6D), which were very stable with only a minority of them vesiculating within the time of observation (Figure S6F). Virtually no cells expressing Endo-K4A4 had tubules or vesicles (Figures 6G and S6D), consistent with its impaired membrane binding ability. Thus, increasing the number of amphipathic helices on a BAR domain increases its ability to induce membrane fission and vesiculation.

Endophilin has a C-terminal SH3 domain (Figure 6E) that can bind to dynamin, which could contribute to the membrane scission observed in vivo. Thus, we assessed the impact of the endophilin mutations on membrane fission in vitro using our biochemical vesiculation assay. We already noted that EndoA3-WT led to approximately 50% vesiculation (Figure 6B). Doubling the length of the N-terminal amphipathic helix led to 80% vesiculation (Figure 6H), consistent with the nanovesicles observed by electron microscopy (Figures 6I and S6I). This correlated very well with an increase in vesicle production in cells (Figure 6G). Vesiculation was decreased for Endo-K8 (Figure 6G). Given that Endo-WT, -K8, and -DAH all bound to membranes to similar extents (Figure 6F), the major consequences on membrane curvature/vesiculation must be a result of the differences in the area occupied by amphipathic helices per scaffold. In conclusion, mutants of endophilin designed to shift it toward the scaffolding or hydrophobic insertion extremes, appear to shift the protein behavior in vitro and in vivo to tubules or vesicles, respectively (Figure 6J). Thus, our experiments on endophilin N-terminal helix mutants show that hydrophobic insertions can not only drive an increase in positive membrane curvature but also help drive membrane scission, likely through destabilizing the membrane neck.

Amphipathic Helix Addition to a BAR Domain Is Sufficient to Mediate Membrane Fission

Finally, to test further whether amphipathic helices could counteract the scaffolding activity of BAR domains, we tested a BAR-domain protein with no known amphipathic helix. Expression of WT β2-centaurin BAR+PH domain (centaurin-WT, Figure 7A) induced extensive tubulation when expressed in cells (Peter et al., 2004) (Figure 7B) and some tubulation of liposomes (Figure 7D). Initially we observed that GRAF and centaurin competes with epsin for liposome vesiculation (Figures 7E and S7), but this effect may be due to competition for binding sites on the membrane. To circumvent this, we added one or two copies of the N-terminal amphipathic helix from endophilin onto centaurin (Figure 7A) and tested the ability of the mutants to induce membrane fission both in vivo and in vitro. Expression of centaurin containing a double amphipathic helix (centaurin-DAH) caused remarkable vesiculation in vitro (Figure 7D) and in cells (Figure 7B), whereas addition of a single amphipathic helix (centaurin-SAH) gave an intermediate phenotype. This further confirmed the prediction of the model that amphipathic helices support membrane scission and that this activity is counteracted by BAR-domain scaffolding.

Experimental Evidence Agrees Quantitatively with the Model

We found a strong positive correlation for vesicle production in vitro and in vivo for the different numbers of amphipathic helices per BAR domain (Figure 6J). Additionally, the experimental data reflected the predictions of our model qualitatively. Epsin ENTH domains having no scaffolding effect, and endophilin-DAH possessing elongated amphipathic helices, are predicted to transform flat membranes directly to the vesicular phase for all system compositions without intermediate generation of a thermodynamically equilibrium tubular phase, although kinetically trapped but nonequilibrium tubules might be observed (Figure S1I for $\kappa_p = 0$). Indeed epsin ENTH domains generated small vesicles without formation of equilibrium membrane tubules (Figure 4), and endophilin-DAH converted the membranes, predominantly, into small spherical vesicles (Figures 6H, 6I, and S6J) with rare tubules. Further, proteins such as centaurin that lack the membrane-inserting modules but have crescent-like scaffolding domains will, according to the model, bend membrane into tubular shapes, but no membrane fission will occur (Figures 1 and S1), which agrees with our experimental observations (Figure 7).

In addition to the qualitative agreement, the model predicted quantitatively the percentage of membrane vesiculation by endophilin mutants with a varying overall area, A_{ins}, occupied in the membrane plane by the amphipathic helices belonging to one BAR domain (Figure 7F). The area A_{ins} is very small for K8 mutant (taken as A_{ins} = 8 nm^2) and was estimated as A_{ins} = 20 nm^2 for endophilin WT and A_{ins} = 32 nm^2 for endophilin-DAH. The theoretical curve (assuming 50% coverage of the membrane surface area by the proteins) along with the results of measurements in the liposome system and in cells are presented in Figure 7F, which shows a good agreement between the model predictions and the experimental results taking account the considerable variations in the latter. Finally, we computed the average radii of vesicles generated by the epsin

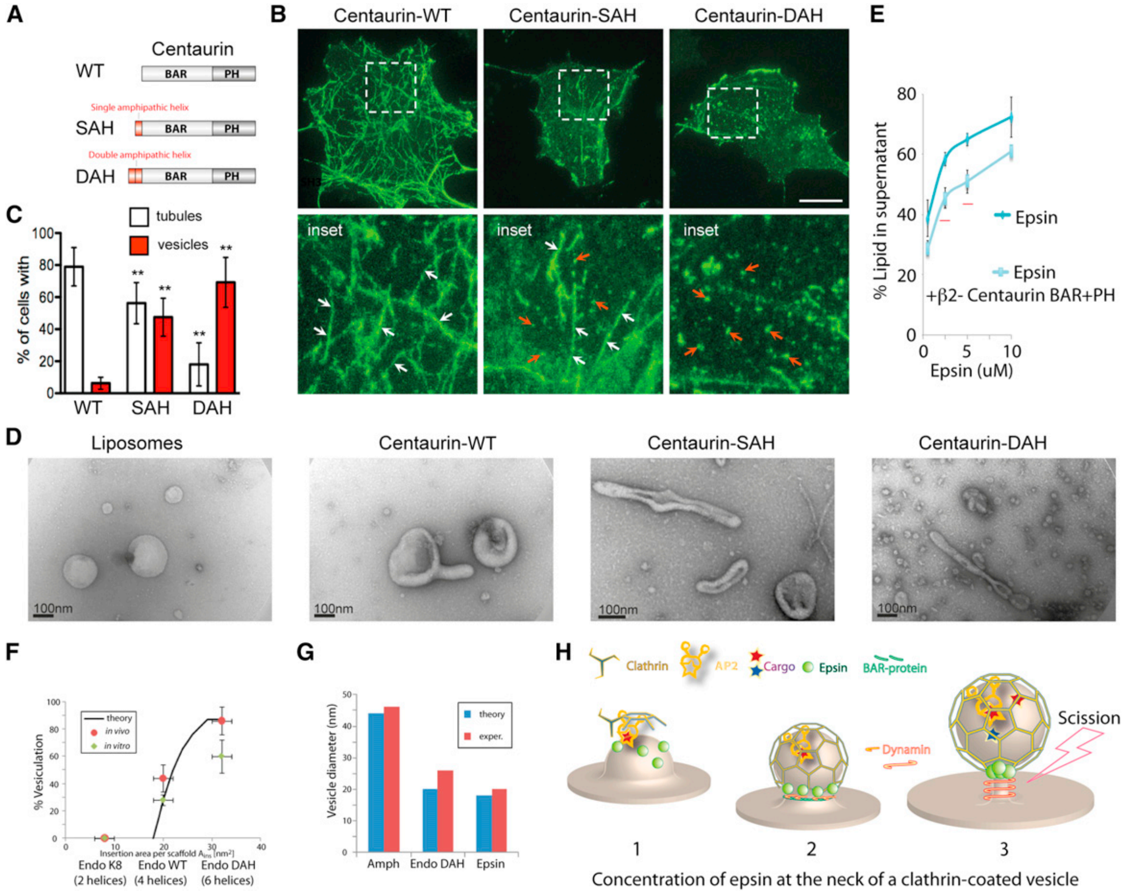

Figure 7. Amphipathic Helix Addition to a BAR Scaffold Is Sufficient to Mediate Membrane Scission

(A) Schematic representation of mutant centaurin proteins. β2-Centaurin WT BAR+PH domain had no amphipathic helices. Centaurin-SAH and centaurin-DAH had respectively a Single Amphipathic Helix or a Double Amphipathic Helix from EndoA3 at their N terminus. All constructs had a Myc-tag at the N terminus.

(B) Confocal images of COS-7 cells expressing the BAR+PH domains of centaurin-WT, centaurin-SAH, or centaurin-DAH. The first row represents the maximal projection of a 3D stack of images acquired at 0.25 μm apart. The second row displays the insets of the boxed regions. Note the tubules (white arrows) and the internal vesicles (red arrows). Bar, 10 μm.

(C) Histogram showing the percentage of transfected cells displaying internal tubules (white) and internal vesicles (red). Cells could present both. Data are the mean ± SD of >300 cells for each constructs from three independent experiments. $^{**}p < 0.001$.

(D) EM of liposomes with 9 μM of the indicated proteins.

(E) Competition between epsin ENTH domain and β2-centaurin for vesiculation/tubulation of Folch liposomes. Mean ± SD for three independent experiments. Red bar: $p < 0.001$.

(F) Predicted percentage of vesiculated membrane by N-BAR domains covering 50% of the total membrane area as a function of the total area of inclusions per scaffold A_{ins}. Points represent the measured values in vitro and in vivo (Figure 6) for Endo-K8 (A_{ins} = 7 nm^2), Endo-WT (A_{ins} = 20 nm^2, and Endo-DAH (A_{ins} = 32 nm^2).

(G) Predicted and measured diameters of vesicles generated as a result of membrane fission by Amph (A_{ins} = 12 nm^2, Endo-DAH (A_{ins} = 32 nm^2), and epsin ENTH domain (A_{ins} = 6 nm^2). In the computations 50% membrane coverage was used.

(H) Model of the concentration of epsin to the region of membrane scission during CCV maturation.

See also Figure S7.

ENTH domains, amphiphysin, and endophilin DAH (again for 50% membrane area occupied by the proteins). There is good agreement (Figure 7G), further validating the model and substantiating our experimental results.

DISCUSSION

Epsin Supports CCV Budding

Epsin proteins are associated with CCS (Chen et al., 1998) and accumulate gradually with peak accrual coinciding with CCV budding (Taylor et al., 2011). In our in vitro assays, epsin ENTH domain was sufficient to drive membrane fission. In cells, clathrin will act as a scaffold on curvature and may even limit the extent of curvature under the cage. It is likely that as clathrin coats mature, epsin molecules get pushed to the edge of the cage, consistent with its nonenrichment in mature CCV (Mills et al., 2003), and with its proposed localization at the neck of the nascent vesicle (Saffarian et al., 2009) where it will largely be unrestrained by clathrin (Figure 7H). Scission of CCV is believed to be primarily carried out by dynamin in higher eukaryotes. We now show

that dynamin-mediated fission of CCVs is severely compromised in the absence of epsin (Figure 2) and that, in certain conditions, epsin can palliate the depletion of dynamin and support CCV budding (Figure 3). However, epsin cannot support budding when dynamin activity is blocked by dynasore and dynamin accumulates at the neck of CCS. The failure of fission may be due to the stabilization of the neck by an oligomeric dynamin scaffold, rather like BAR scaffolds. Altogether, this suggests that epsin might provide the required force to destabilize the neck of nascent vesicles and that scaffolding generated by dynamin oligomers might act as a ‘‘timer’’ with membrane fission promoted upon cooperative GTP hydrolysis-mediated depolymerization.

Shallow Hydrophobic Insertions Promote and BAR-Domain Scaffolds Restrain Membrane Fission

Compared to BAR scaffolds alone, N-BAR modules (e.g., endophilin, amphiphysin) contain additional amphipathic helices that insert into membranes. Amphiphysin and endophilin both are recruited to endocytic spots with dynamin (Taylor et al., 2011). Hydrophobic insertions are likely to enable these BAR proteins to create the neck and may further position them on the pathway to membrane scission. For dynamin-independent pathways we speculate hydrophobic insertions will be a major driving force for membrane scission. At sufficient concentrations many different proteins with insertions may contribute to membrane fission, or curvature may be limited by associated scaffolds. For example, Arf and Sar proteins contribute to the formation of COP coated vesicles (Beck et al., 2011; Lee et al., 2005). Because both Arf and Sar have an amphipathic helix that extends upon GTP binding, it is likely that these proteins contribute to the scission reaction by the hydrophobic insertion mechanism. For these proteins, the effects of amphipathic helix insertion may initially be controlled by the COP coat, just as the effects of epsin will be limited by the clathrin coat (Figure 7H), perhaps controlling the timing of membrane fission. Arf proteins are also known to bind to the BAR-domain protein, arfaptin (Williger et al., 1999), suggesting a potential regulation of the extent of curvature produced, which could well lead to vesicle budding. Finally, budding of some viruses also relies on amphipathic helix insertion, such as the M2 protein of influenza virus (Rossman et al., 2010). We will likely discover many more examples as the importance of hydrophobic insertions in membrane fission is recognized.

EXPERIMENTAL PROCEDURES

A full description of the methods is in the Extended Experimental Procedures.

Cell Culture, RNAi, Live-Cell Imaging, and Ligand Uptake Measurement by Flow Cytometry

HeLa, BSC1, hTERT-RPE1, COS-7, and BSC1 stably expressing σ2-EGFP, SK-MEL-2 DNM2^{en-all}-EGFP, and DNM2^{en-all}-EGFP CLTA^{en-all}-RFP genome-edited (Doyon et al., 2011) cells were grown on 35 mm glass-bottom dishes (MatTek, imaging) or 100 mm dishes (ligand uptake). RNAi was carried out by double transfection (on days 1 and 2) with oligofectamine (Invitrogen) and 80 pmol of each indicated siRNA (see Extended Experimental Procedures) and analyzed on day 3. It is important to note that efficient knockdown of CHC, AP2, FCHo1+2, DNM1+2, and Epsin1+2+3 induce extensive cell mortality and that effectively knocked down cells are often in a minority (see Figure S2D). AlexaFluor 488-labeled human transferrin (20 μg/ml) and FITC-labeled Dextran 3000 kDa (1 mg/ml) uptake was carried at 37°C for 7 and 15 min, respectively, and analyzed using LSR II flow cytometer (Becton Dickinson). Live-cell imaging was performed as in Henne et al. (2010).

Liposome Preparation, Binding, and Vesiculation

Purified untagged proteins and Folch liposomes spiked with 5% PIP2 were used in the experiments, unless otherwise indicated. Folch liposomes (50:50 mix of Sigma Aldrich[B-1502]):Avanti Polar Lipids(131101P) with 0%–5% PI(4,5)P_2 (Avanti Polar Lipids, 840046P) in 100 mM NaCl, 20 mM HEPES (pH 7.4) were extruded seven times through 200 nm polycarbonate membranes (Nuclepore). For tubulation/vesiculation assays 5 or 10 μl of 1 mg/ml liposomes were used in 40 μl reactions. Samples were spread on glow-discharged electron microscopy grids (Agar Scientific) and stained using 2% uranyl acetate.

Biochemical Membrane Fission Assay

Liposomes as above were incubated with protein for 1 hr at room temperature (although much shorter times can be used) and were spun at 250,000 × *g* for 15 min in a Beckman TLA100 rotor. Resuspended pellets and supernatants were analyzed by SDS-PAGE. We monitored the distribution of both proteins and lipids by SDS-PAGE using Bis-Tris gels run in MES buffer (to avoid excess counterions at the gel front that interfere with lipid staining). Gels were stained with 0.1% Coomassie in 10% acetic acid for 5 min and then destained in water. Alcohol was avoided in order to not solubilize the lipids in the gels. Loading dye (Bromophenol Blue) can interfere with the quantitation and so at least 30 min was given for this dye to leach from the gel. The extent of vesiculation was measured as the percentage of lipid found in the supernatant. This is a slight underestimation as empty lanes have a background that has not been subtracted as this can vary across the gel. Gels were quantitated using ImageJ.

Statistical Analysis

Results are mean ± standard deviation (SD) or median with interquartile range, as indicated. Significance was calculated using the Student's t test.

SUPPLEMENTAL INFORMATION

Supplemental Information includes Extended Experimental Procedures and seven figures and can be found with this article online at doi:10.1016/j.cell.2012.01.047.

ACKNOWLEDGMENTS

We thank Sew Yeu Peak-Chew for Mass Spectrometry, Tom Kirchhausen and David Drubin for the kind gift of σ2-EGFP BSC1 cells and SK-MEL-2 genome-edited cells, respectively, Rohit Mittal for helpful comments, and Felix Campelo for a supplemental computation. This work was supported by the Israel Science Foundation (ISF) and Marie Curie Network ‘‘Virus Entry’’ (M.M.K.), the Medical Research Council UK (grant number U105178795) (H.T.M., E.E., E.B.), Cambridge Overseas Trust and Trinity College Cambridge (G.C.), and Austrian Academy Science Fellowship (N.L.). E.B. designed, performed, and quantified live and fixed-cell imaging and ligand uptake, A.P. did theoretical modeling, G.C. and N.L. purified proteins and performed vesiculation assay and electron microscopy, E.E. performed and quantified electron microscopy of clathrin-coated pits, H.T.M. designed and performed vesiculation assay and supervised the project, M.M.K. suggested the mechanism and supervised the theoretical modeling. H.T.M., M.M.K., and E.B. wrote the paper with input from all the other authors.

Received: May 16, 2011
Revised: November 9, 2011
Accepted: January 5, 2012
Published: March 29, 2012

REFERENCES

Antonny, B. (2006). Membrane deformation by protein coats. Curr. Opin. Cell Biol. *18*, 386–394.

Bashkirov, P.V., Akimov, S.A., Evseev, A.I., Schmid, S.L., Zimmerberg, J., and Frolov, V.A. (2008). GTPase cycle of dynamin is coupled to membrane squeeze and release, leading to spontaneous fission. Cell *135*, 1276–1286.

Beck, R., Prinz, S., Diestelkötter-Bachert, P., Röhling, S., Adolf, F., Hoehner, K., Welsch, S., Ronchi, P., Brügger, B., Briggs, J.A., and Wieland, F. (2011). Coatomer and dimeric ADP ribosylation factor 1 promote distinct steps in membrane scission. J. Cell Biol. *194*, 765–777.

Campelo, F., McMahon, H.T., and Kozlov, M.M. (2008). The hydrophobic insertion mechanism of membrane curvature generation by proteins. Biophys. J. *95*, 2325–2339.

Chen, C., and Zhuang, X. (2008). Epsin 1 is a cargo-specific adaptor for the clathrin-mediated endocytosis of the influenza virus. Proc. Natl. Acad. Sci. USA *105*, 11790–11795.

Chen, H., Fre, S., Slepnev, V.I., Capua, M.R., Takei, K., Butler, M.H., Di Fiore, P.P., and De Camilli, P. (1998). Epsin is an EH-domain-binding protein implicated in clathrin-mediated endocytosis. Nature *394*, 793–797.

Chen, H., Ko, G., Zatti, A., Di Giacomo, G., Liu, L., Raiteri, E., Perucco, E., Collesi, C., Min, W., Zeiss, C., et al. (2009). Embryonic arrest at midgestation and disruption of Notch signaling produced by the absence of both epsin 1 and epsin 2 in mice. Proc. Natl. Acad. Sci. USA *106*, 13838–13843.

Chernomordik, L.V., and Kozlov, M.M. (2003). Protein-lipid interplay in fusion and fission of biological membranes. Annu. Rev. Biochem. *72*, 175–207.

Coon, B.G., Direnzo, D.M., Konieczny, S.F., and Aguilar, R.C. (2011). Epsins' novel role in cancer cell invasion. Commun Integr Biol *4*, 95–97.

Corda, D., Colanzi, A., and Luini, A. (2006). The multiple activities of CtBP/BARS proteins: the Golgi view. Trends Cell Biol. *16*, 167–173.

Doyon, J.B., Zeitler, B., Cheng, J., Cheng, A.T., Cherone, J.M., Santiago, Y., Lee, A.H., Vo, T.D., Doyon, Y., Miller, J.C., et al. (2011). Rapid and efficient clathrin-mediated endocytosis revealed in genome-edited mammalian cells. Nat. Cell Biol. *13*, 331–337.

Farsad, K., and De Camilli, P. (2003). Mechanisms of membrane deformation. Curr. Opin. Cell Biol. *15*, 372–381.

Ford, M.G., Pearse, B.M., Higgins, M.K., Vallis, Y., Owen, D.J., Gibson, A., Hopkins, C.R., Evans, P.R., and McMahon, H.T. (2001). Simultaneous binding of PtdIns(4,5)P2 and clathrin by AP180 in the nucleation of clathrin lattices on membranes. Science *291*, 1051–1055.

Ford, M.G., Mills, I.G., Peter, B.J., Vallis, Y., Praefcke, G.J., Evans, P.R., and McMahon, H.T. (2002). Curvature of clathrin-coated pits driven by epsin. Nature *419*, 361–366.

Gallop, J.L., Jao, C.C., Kent, H.M., Butler, P.J., Evans, P.R., Langen, R., and McMahon, H.T. (2006). Mechanism of endophilin N-BAR domain-mediated membrane curvature. EMBO J. *25*, 2898–2910.

Goormaghtigh, E., and Scarborough, G.A. (1986). Density-based separation of liposomes by glycerol gradient centrifugation. Anal. Biochem. *159*, 122–131.

Henne, W.M., Boucrot, E., Meinecke, M., Evergren, E., Vallis, Y., Mittal, R., and McMahon, H.T. (2010). FCHo proteins are nucleators of clathrin-mediated endocytosis. Science *328*, 1281–1284.

Hughes, A.C., Errington, R., Fricker-Gates, R., and Jones, L. (2004). Endophilin A3 forms filamentous structures that colocalise with microtubules but not with actin filaments. Brain Res. Mol. Brain Res. *128*, 182–192.

Hurley, J.H., and Hanson, P.I. (2010). Membrane budding and scission by the ESCRT machinery: it's all in the neck. Nat. Rev. Mol. Cell Biol. *11*, 556–566.

Huse, D.A., and Leibler, S. (1991). Are sponge phases of membranes experimental gauge-Higgs systems? Phys. Rev. Lett. *66*, 437–440.

Jakobsson, J., Gad, H., Andersson, F., Löw, P., Shupliakov, O., and Brodin, L. (2008). Role of epsin 1 in synaptic vesicle endocytosis. Proc. Natl. Acad. Sci. USA *105*, 6445–6450.

Kazazic, M., Bertelsen, V., Pedersen, K.W., Vuong, T.T., Grandal, M.V., Rødland, M.S., Traub, L.M., Stang, E., and Madshus, I.H. (2009). Epsin 1 is involved in recruitment of ubiquitinated EGF receptors into clathrin-coated pits. Traffic *10*, 235–245.

Kozlovsky, Y., and Kozlov, M.M. (2003). Membrane fission: model for intermediate structures. Biophys. J. *85*, 85–96.

Kweon, D.H., Shin, Y.K., Shin, J.Y., Lee, J.H., Lee, J.B., Seo, J.H., and Kim, Y.S. (2006). Membrane topology of helix 0 of the Epsin N-terminal homology domain. Mol. Cells *21*, 428–435.

Lee, M.C., Orci, L., Hamamoto, S., Futai, E., Ravazzola, M., and Schekman, R. (2005). Sar1p N-terminal helix initiates membrane curvature and completes the fission of a COPII vesicle. Cell *122*, 605–617.

Liu, J., Sun, Y., Drubin, D.G., and Oster, G.F. (2009). The mechanochemistry of endocytosis. PLoS Biol. *7*, e1000204.

Lundmark, R., Doherty, G.J., Howes, M.T., Cortese, K., Vallis, Y., Parton, R.G., and McMahon, H.T. (2008). The GTPase-activating protein GRAF1 regulates the CLIC/GEEC endocytic pathway. Curr. Biol. *18*, 1802–1808.

McMahon, H.T., and Gallop, J.L. (2005). Membrane curvature and mechanisms of dynamic cell membrane remodelling. Nature *438*, 590–596.

McMahon, H.T., and Boucrot, E. (2011). Molecular mechanism and physiological functions of clathrin-mediated endocytosis. Nat. Rev. Mol. Cell Biol. *12*, 517–533.

Mills, I.G., Praefcke, G.J., Vallis, Y., Peter, B.J., Olesen, L.E., Gallop, J.L., Butler, P.J., Evans, P.R., and McMahon, H.T. (2003). EpsinR: an AP1/clathrin interacting protein involved in vesicle trafficking. J. Cell Biol. *160*, 213–222.

Peter, B.J., Kent, H.M., Mills, I.G., Vallis, Y., Butler, P.J., Evans, P.R., and McMahon, H.T. (2004). BAR domains as sensors of membrane curvature: the amphiphysin BAR structure. Science *303*, 495–499.

Rossman, J.S., Jing, X., Leser, G.P., and Lamb, R.A. (2010). Influenza virus M2 protein mediates ESCRT-independent membrane scission. Cell *142*, 902–913.

Roux, A., Uyhazi, K., Frost, A., and De Camilli, P. (2006). GTP-dependent twisting of dynamin implicates constriction and tension in membrane fission. Nature *441*, 528–531.

Saffarian, S., Cocucci, E., and Kirchhausen, T. (2009). Distinct dynamics of endocytic clathrin-coated pits and coated plaques. PLoS Biol. *7*, e1000191.

Schmid, S.L., and Frolov, V.A. (2011). Dynamin: functional design of a membrane fission catalyst. Annu. Rev. Cell Dev. Biol. *27*, 79–105.

Schwarz, U.S., and Gompper, G. (1999). Systematic approach to bicontinuous cubic phases in ternary amphiphilic systems. Phys. Rev. E Stat. Phys. Plasmas Fluids Relat. Interdiscip. Topics *59*(*5 Pt B*), 5528–5541.

Shibata, Y., Hu, J., Kozlov, M.M., and Rapoport, T.A. (2009). Mechanisms shaping the membranes of cellular organelles. Annu. Rev. Cell Dev. Biol. *25*, 329–354.

Taylor, M.J., Perrais, D., and Merrifield, C.J. (2011). A high precision survey of the molecular dynamics of mammalian clathrin-mediated endocytosis. PLoS Biol. *9*, e1000604.

Wang, J., Gossing, M., Fang, P., Zimmermann, J., Li, X., von Mollard, G.F., Niu, L., and Teng, M. (2011). Epsin N-terminal homology domains bind on opposite sides of two SNAREs. Proc. Natl. Acad. Sci. USA *108*, 12277–12282.

Williger, B.T., Provost, J.J., Ho, W.T., Milstine, J., and Exton, J.H. (1999). Arfaptin 1 forms a complex with ADP-ribosylation factor and inhibits phospholipase D. FEBS Lett. *454*, 85–89.

EXTENDED EXPERIMENTAL PROCEDURES

Cloning and Protein Purification

Full-length rat amphiphysin 2-6 (Wigge et al., 1997) and full-length rat endophilin A1 were cloned into pGEX-6P2. Full-length rat epsin 1 WT, L6E and L6W (Ford et al., 2002) and full-length human endophilin A3 WT, K4A4, K8 and DAH were cloned into the Gateway system and expressed with a C-terminal GST tag for protein purification and with a C-terminal Tag-RFP-T (Shaner et al., 2008) or EGFP tag for in vivo expression. Human centaurinβ2 BAR+PH domain WT or fused with one (SAH) or two (DAH) copies of the N-terminal amphipathic helix of human endophilin A3 were cloned into pGEX-4T2 or cloned into a CMV vector with an N-terminal Myc tag for in vivo detection. The ENTH domain of rat epsin1 (residues 1-164) and its L6E or L6W variants were cloned into pGEX-4T2. Proteins were expressed in BL21 cells for 1 hr at 37°C for amphiphysin, 16 hr at 18°C for the remaining proteins. Cells were lysed using Emulsiflex C3, spun at 140,000xg for 40 min at 4°C in a Beckman Ti45 rotor, and the supernatant was bound to glutathione beads for 30 min. The beads were washed extensively with 150 mM NaCl, 20 mM HEPES, pH 7.4, 2 mM DTT, 2 mM EDTA, with 2 washes at 500 mM NaCl in between. The GST tag was cleaved using PreScission or Thrombin proteases, and cleaved proteins were further purified by Superdex 200 gel filtration.

Cell Culture, RNAi, and Ligand Uptake Measurement by Flow Cytometry

HeLa cells (ECACC 93021013), BSC1 (ECACC 85011422), COS-7 (ATCC CRL-1651), BSC1 stably expressing σ2-EGFP (Ehrlich et al., 2004), were cultured in DMEM-GlutaMAX-I media (GIBCO) supplemented with 10% (v/v) fetal bovine serum (FBS). hTERT-RPE1 (ATCC CRL-4000) and SK-MEL-2 DNM2$^{en\text{-}all}$-EGFP and DNM2$^{en\text{-}all}$-EGFP CLTA$^{en\text{-}all}$-RFP genome-edited (Doyon et al., 2011) were cultured in DMEM:F12 HAM (1:1 v/v), 0.25% sodium bicarbonate (w/v), 1 mM GlutaMax and 10% FBS.

Approximately 6 x 10^5 HeLa, and 1 x 10^6 BS-C-1 and hTERT-RPE1 cell grown in 100 mm dishes were transfected twice (on days 1 and 2) with oligofectamine (Invitrogen) with a total of 600 pmol of the indicated siRNA and analyzed on day 4 (72 hr after the first transfection). The siRNA used were: Epsin 1+2+3 pool 1: HSS121071 (2 oligos against human epsin1), HSS117872 (2 oligos against human epsin 2), and HSS147867 (2 oligos against epsin 3) (Invitrogen); Epsin 1+2+3 pool 2: J-004724-06, J-004725-06 and J-021006-06 (Thermo Scientific, one oligo per reference); Epsin 1+2+3 pool 3: HSS121069, HSS177016 and HSS147865 (Invitrogen, 2 oligos per reference); Epsin 1+2+3 pool 4: HSS121070, HSS177871 and HSS147866 (Invitrogen, 2 oligos per reference) and epsin 1+2+3 pool 5: J-004724-05, J-004725-05 and J-021006-05 (Thermo Sientific, 1 oligo per reference). Other siRNA used: Clathrin Heavy chain (CHC): HSS174637 (Invitrogen), AP2: mu2-2 defined in Motley et al. (2003), FCHo1+2: HSS118257 and HSS151016 (Invitrogen, 2 and 1 oligos per reference, respectively), DNM1+2: HSS176208 and J-004007-06 and J-004007-08 (Invitrogen and Thermo Scientific, respectively). Control samples were transfected in the same way than the RNAi samples but a scrambled control siRNA oligo (Invitrogen) was used instead. Rescue by rat epsin1-RFP WT, L6W and L6E was performed by cotransfection (with siRNA) of 10 ng of DNA per 2 x 10^4 cells on days 1 and 2 using Fugene 6 (HeLa cells) or Lipofectamine2000 (BSC1 and hTERT-RPE1 cells).

It is important to note that efficient knockdown of CHC, AP2, FCHO1+2, DNM1+2, and Epsin1+2+3 induces significant cell mortality and that the cells fully knocked-down are often in minority (see Figure S2D). We also noted that splitting cells after the first siRNA transfection reduced drastically the number of cells with the strongest decreases in transferrin uptake (not shown). Consistent with a role for clathrin-mediated endocytosis in cell adhesion (Ezratty et al., 2009), it is likely that efficient blockage of clathrin-mediated endocytosis by CHC, AP2, FCHO1+2 or Epsin1+2+3 RNAi perturb the cells to readhere after detachment during cell splitting.

AlexaFluor 488-labeled human transferrin (Molecular Probes, used at 20 μg/ml) and FITC-labeled Dextran 3000 kDa (Molecular Probes, used at 1mg/ml) uptake was carried at 37°C for 7 and 15 min, respectively. The dynamin small inhibitor dynasore (Macia et al., 2006) was used at 80 μM (in 0.1% serum medium) for 20 min before ligand addition or live-cell imaging. Similar phenotypes were obtained with 15 and 60 min incubation, respectively (not shown). Cells were then washed with ice-cold PBS, detached by 1 min incubation with 0.25% Trypsin-EDTA, spun, acid-washed (ice-cold buffer, pH 5.5; to remove surface-bound ligand), washed, fixed (PFA 3.7% for 20 min), washed, and resuspended in PBS and analyzed using LSR II flow cytometer (Beckson-Dickinson). Flow cytometry provided similar phenotypes than those obtained by classical microscopy-based measurement of ligand uptake but allowed an increase in at least 2 Log in the number of cell analyzed.

Cell Culture, Transfection, and Live-Cell and Fixed-Cell Fluorescent Microscopy

Approximately 2 x 10^5 or 2.5 x 10^4 cells were cultured on 35mm glass bottom dishes (MatTek) or 13 mm coverslip, respectively. Cells were transfected using FuGene 6 (Roch Diagnostics) or Lipofectamine2000 (Invitrogen) using 0.05 to 1 μg of the various plasmids. Cells were incubated 24 hr to express the constructs before imaging. RNAi for imaging experiments was carried by double transfection (on days 1 and 2) with oligofectamine (Invitrogen) and a total of 80 pmol of each indicated siRNA (see details above) and analyzed on day 4 (72 hr after the first transfection).

Cells were imaged live directly (for EGFP and TagRFP-T constructs) or fixed (3.7% paraformaldehyde, 20 min, room temperature) and stained using rabbit anti-Myc (2272 Cell signaling) and goat anti-Rabbit Alexa 488 (Molecular Probes) and mounted on slides using Dabco. Just before live-cell imaging, the medium was changed to MEM without phenol red supplemented with 20 mM HEPES, pH 7.4, and 5% FBS and placed into a temperature controlled chamber on the microscope stage with 95% air:5% CO_2 and 100%

humidity. FM4-64 dye (Molecular Probes) was used at 5 μg/ml in imaging medium. The cells were incubated for 1 min with the dye, washed once and a chase of 10 min was performed in fresh imaging medium to allow the dye to be internalized into the endocytic network.

Live-cell and fixed-cell imaging data were acquired using a fully motorized inverted microscope (Eclipse TE-2000, Nikon) equipped with a CSU-X1 spinning disk confocal head (UltraVIEW VoX, Perkin-Elmer, England) using a 60x lens (Plan Apochromat VC, 1.4 NA, Nikon) under control of Volocity 5.0 (Improvision, England). 14-bit digital images were obtained with a cooled EMCCD camera (9100-02, Hamamatsu, Japan). Two 50 mW solid-state lasers (488 and 561; Crystal Laser and Melles Griots) coupled to individual acoustic-optical tunable filter (AOTF) were used as light source to excite EGFP, Alexa 488, and FM4-64, as appropriate. Live-cell imaging was performed as in Henne et al. (2010) and analyzed and presented according to Boulant et al. (2011).

Electron Microscopy

BSC-1 cells treated with Epsin1+2+3 RNAi (pool 1) were sorted in a Beckman Coulter MoFlo cell sorter after Alexa 488 Transferrin uptake. Knockdown and control cells were fixed in 2.5% glutaraldehyde, 2% paraformaldehyde, 2% tannic acid, 0.1M cacodylic acid, pH 7.4. After postfixation in 1% osmium tetraoxide, the cells were dehydrated in a graded series of ethanol and embedded in Durcupan resin (Fluka). Ultrathin serial sections (70 nm) were counterstained and viewed in a Philips 208 80 kV electron microscope. For the morphometric analysis cells with an intact perimeter were photographed at a low magnification and the number of clathrin-coated structures connected to the plasma membrane were counted.

Liposome Preparation, Binding, and Vesiculation

Folch liposomes (50:50 mix of Sigma Aldrich[B-1502]:Avanti Polar Lipids[131101P] with 0%–5% PI(4,5)P_2 [Avanti Polar Lipids, 840046P]) in 100 mM NaCl, 20 mM HEPES, pH 7.4 were extruded 7 times through 200 nm polycarbonate membranes (Nuclepore). For tubulation/vesiculation assays 5 or 10 μl of 1 mg/ml liposomes were used in 40 μl reactions. Samples were spread on glow-discharged electron microscopy grids (Agar Scientific) and stained using 2% uranyl acetate and viewed in a Philips 208 80 kV electron microscope. Negative staining EM quantitation: Vesicle number and size was measure in micrographs from at least 3 different fields of view using Adobe Illustrator. Sizes were binned and graphed in Excel.

Biochemical Membrane Fission Assay

Liposomes as above were incubated with protein for 60 min at room temperature (although much shorter times can also be used) and were spun at 250,000xg for 15 min in a Beckman TLA100 rotor. Resuspended pellets and supernatants were analyzed by SDS-PAGE. In contrast to the electron microscopy assay, this allowed us to look at the global effect on liposomes, in a more quantitative manner. We monitored the distribution of both proteins and lipids by SDS PAGE using Bis-Tris gels run in MES buffer (to avoid excess counter ions at the gel front which interfere with lipid staining). The gels were stained with 0.1% Coomassie in 10% acetic acid for 5 min and then destained in water. Alcohol was avoided in order to not solubilize the lipids in the gels. Loading dye (Bromophenol Blue) can interfere with the quantitation and so at least 30 min was given for this dye to leach from the gel. The extent of vesiculation was measured as the % lipid found in the supernatant. This is a slight underestimation as empty lanes have a background that has not been subtracted as this can vary across the gel. Gels were quantitated using ImageJ.

Limited Proteolysis of Epsin ENTH Domain Amphipathic Helix

Trypsin (5 ng) was incubated with epsin ENTH domain (10 μmol) for 20 min at 27°C. The reaction was terminated by addition of 100 ng of soybean trypsin inhibitor.

Mass Spectrometry

Epsin ENTH domain (10 mM) was diluted to 2.5 mM in water and digested with trypsin for 4 hr. 3 μl of the sample was diluted to 30 μl with 2% formic acid and desalted with C18 Zip-Tip (Millipore). The peptides were eluted from the Zip-Tip with 5 μl of 60% MeCN/2% formic acid. 0.7μl of peptides wad spotted onto Maldi target followed by 0.7 μl of saturated DHB (2,5-dihydroxybenzoic acid in 10% MeCN/ 0.1% TFA). All peptide MW measurements were carried out on an Ultraflex III mass spectrometer (Bruker, Bremen).

Theoretical Modeling

Physical Model and Main Equations

To substantiate quantitatively the proposed mechanism of membrane fission by hydrophobic insertions and its limitation by the crescent-like protein scaffolds, we developed a simple theoretical model. We considered an initially flat continuous lipid bilayer and asked how this bilayer would be shaped upon shallow insertion of amphipathic helices into its outer monolayer and attachment of arc-like protein scaffolds to its outer surface (Figures S1A–S1C). We assumed that the insertions and scaffolds are uniformly distributed all along the outer surface of the membrane, which means that the membrane has to be, on average, homogeneously bent. This is possible if the membrane adopts either a cylindrical shape of a membrane tubule or a spherical shape of a membrane vesicle. While to form a tubule of any specific curvature, the initial bilayer has to be merely bent without any changes of its continuity, formation of vesicles of a certain radius (curvature) requires also scission of the initial continuous membrane into separate membranes of areas determined by the vesicle radius. Hence, vesicle formation involves membrane fission in addition to curvature generation. The states

of the system resulting from the membrane shaping by proteins into a tubule or vesicles will be referred below to as the tubular and vesicular phases, respectively.

Method

Our goal is to determine the stable conformation of the lipid bilayer upon action of hydrophobic insertions and crescent-like protein scaffolds at different protein-to-lipid ratios and to find the conditions of transition between the tubular and vesicular phases. We employ the standard thermodynamic method of common tangents commonly used to analyze the phase behavior of mixed systems (see e.g., Hillert, 1998).

We characterize the system composition by the molar ratio

$$x = N_p / N_l, \tag{1}$$

where N_p and N_l are, respectively, the numbers of the protein and lipid molecules in the membrane. The free energies of the tubular and vesicular phases can be expressed, respectively, as $F^c = N_l^c \cdot g^c(x^c)$ and $F^s = N_l^s \cdot g^s(x^s)$, where $g(x)$ is the free energy related to one lipid molecule, and the superscripts "c" and "s" denote belonging to the tubular (cylindrical) and vesicular (spherical) phases, respectively. The molar ratios of the tubular and vesicular phases, $x^{c*}$and x^{s*}, corresponding to the system transition between the two phases and referred to as the critical molar ratios, represent the solution of the equations

$$\frac{dg^c(x^c)}{dx^c} = \frac{dg^s(x^s)}{dx^s} \text{ and } g(x^c) - x^c\frac{dg^c(x^c)}{dx^c} = g^s(x^s) - x^s\frac{dg^s(x^s)}{dx^s}. \tag{2}$$

Graphically, this solution is given by the two points where the functions $g^c(x^c)$ and $g^s(x^s)$ are touched by the common tangent represented by dashed lines in Figures S1D–S1F. Thermodynamically, the critical compositions x^{c*} and x^{s*} correspond to equality of the lipid and protein chemical potentials in the two phases. In case the equations (Equation 2) do not have a solution, the stable phase is that with the smallest energy per lipid molecule $g(x)$.

In case, the total protein-to-lipid ratio x^{tot} has an intermediate value between $x^{c*}$and x^{s*}, the two phases coexist each having the critical composition, whereas the total amount of the lipid and protein material is shared between the phases according to the lever rule.

Note that if the function $g(x)$ has a nonmonotonic character with two or more minima, the "internal" common tangents have to be drawn between these minima. These "internal" common tangents correspond to the internal phase separations within the phase described by $g(x)$. In our case, for some parameter ranges, such an internal phase separation within the tubular phase is predicted between the narrow cylinders containing most of the N-BAR domains and the practically flat cylinders with vanishing protein concentration. In cases where the internal common tangents exist, the portions of the curve $g(x)$ located above them have to be replaced by the internal common tangents themselves. This eliminates the concave portions of $g(x)$ corresponding to the unstable states of the system (Hillert, 1998).

In the following, we formulate the models for the free energies of the tubular and vesicular phases, $g^c(x^c)$ and $g^s(x^s)$. The resulting phase diagrams of the system are presented in Figures 1 and S1D–S1F.

Energy of the System

The contributions to the free energy determining the system transition between the tubular and vesicular phases and, hence, relevant for our analysis are the bending energy of the membrane with embedded hydrophobic insertions, f_m, the bending energy of the protein scaffolds, f_B, and the contributions of the translational entropy of proteins on the membrane surface, f_{ent}. In addition, we consider the energy of the steric repulsion between the BAR scaffolds, f_{ster}, which will be relevant only for the cases of an almost full coverage of the membrane surface by the proteins.

The system composition will be characterized, in addition to the protein-to-lipid molar ratio, x, by the area fraction of the hydrophobic insertions in the outer monolayer, ϕ, and the membrane area fraction covered by the proteins, ϕ_A. We denote by A_l the area per one lipid molecule, by A_{ins} the area occupied in the membrane plane by the insertions belonging to one protein, and by A_p the effective membrane area covered by one protein. The latter is determined by the hydrophilic part of the protein such as the BAR scaffold in case of N-BAR domains and has a meaning of the "excluded area." Taking into account (Equation 1) and the fact that the protein-lipid molar ratio in the outer monolayer is $2x$, the insertion area fraction is

$$\phi = \frac{2x \cdot A_{ins}}{A_l + x \cdot A_{ins}}, \tag{3}$$

and the membrane area fraction covered by the proteins is

$$\phi_A = \frac{2x \cdot A_p}{A_l + x \cdot A_{ins}}.$$

In the following, we describe the energies of the membrane with insertions and of the protein scaffolds by a coarse-grained model that accounts effectively rather than specifically for the molecular features. This is an alternative to a numerical simulation approach that would address the system in molecular or even atomic details. However, a coarse-grained description we use is more suitable for a semiquantitative understanding of the expected effects, which is the goal of the present analysis.

Membrane Bending Energy

The bending energy per unit area of the membrane bilayer is given by Helfrich model (Helfrich, 1973)

$$f_m = \frac{1}{2}\kappa_B\left(J - J_s^b\right)^2 + \bar{\kappa}_B K, \tag{4}$$

where J and K are, respectively, the total and Gaussian curvatures of the surface (Spivak, 1970), κ_B and $\bar{\kappa}_B$ are the bending modulus and the modulus of the Gaussian curvature of the membrane, and J_s^b is the membrane spontaneous curvature. We assume that the membrane spontaneous curvature J_s^b is generated only by the insertions in the outer monolayer (Campelo et al., 2008) and is given by

$$J_s^b = \frac{1}{2}\left(0.99\phi^2 + 0.67\phi\right)\ \left[\text{nm}^{-1}\right]. \tag{5}$$

This expression follows from the numerical modeling using a continuous description of the monolayer interior (Campelo et al., 2008). Although more microscopic models may give a somewhat different result, the dependence (Equation 5) is sufficiently exact for a semiquantitative model.

We assume that the bilayer bending rigidity κ_B is the sum of the bending rigidities of the outer and inner monolayers, $\kappa_B = \kappa_m^{out} + \kappa_m^{in}$. The bending rigidity of the inner monolayer will be denoted by $\kappa_m^{in} = \kappa_m$ and its value taken to be $\kappa_m = 10k_BT$ (where k_BT is the product of the Boltzmann constant and the absolute temperature; see e.g., Niggemann et al., 1995). The bending rigidity of the outer monolayer κ_m^{out} depends on the insertion area fraction and can be represented by $1/\kappa_m^{out} = \phi/\kappa_{inc} + (1-\phi)/\kappa_m$, where κ_{inc} is the effective rigidity of the insertion (see e.g., Kozlov and Helfrich, 1992). Assuming the insertions to be much more rigid than the lipids, $\kappa_{inc} \gg \kappa_m$, we obtain

$$\kappa_B = \frac{2-\phi}{1-\phi}\kappa_m. \tag{6}$$

The bilayer modulus of Gaussian curvature $\bar{\kappa}_B$ can be presented as a sum of contributions of the two membrane monolayers,

$$\bar{\kappa}_B = \bar{\kappa}_m^{out} + \bar{\kappa}_m^{in} - 2\kappa_m^{out}J_S^{out}\delta - 2\kappa_m^{in}J_S^{in}\delta, \tag{7}$$

where $\bar{\kappa}_m^{out}$ and $\bar{\kappa}_m^{in}$ are the outer and inner monolayer moduli of Gaussian curvature, J_S^{out} and J_S^{in} are the outer and inner monolayer spontaneous curvatures and δ is the monolayer thickness which is, typically, about 1.5 nm (see, e.g., Siegel and Kozlov, 2004 for the way to obtain this relationship and a result for the case of $J_S^{out} = J_S^{in}$). Each of the monolayer elastic characteristics can be presented as a sum of a background value determined solely by lipids and a contribution coming from the membrane insertions.

The background bilayer modulus of Gaussian curvature, $\bar{\kappa}_B^0$, can be expressed through the background values of the monolayer modulus of Gaussian curvature, $\bar{\kappa}_0$, and of the monolayer spontaneous curvature, J_S^0. Assuming that the lipid related properties of the two monolayers are similar, one obtains

$$\bar{\kappa}_B^0 = 2\bar{\kappa}_0 - 4\kappa_m J_S^0\delta. \tag{8}$$

The value of $\bar{\kappa}_0$ is largely unknown but general considerations predict it to be negative (Templer et al., 1998). Estimations based on membrane fusion data gives for a representative membrane phospholipid DOPC (dioleoylphosphatidylcholine) an approximate value of $\bar{\kappa}_0 = -0.3\ \kappa_m$ (Kozlovsky et al., 2004). For other lipids the estimated values of $\bar{\kappa}_0$ were somewhat more negative (Kozlovsky et al., 2004; Siegel and Kozlov, 2004; Templer et al., 1998). The background monolayer spontaneous curvature J_S^0 depends on the monolayer lipid composition and can, in general, adopt negative as well as positive values. For the sake of our semiquantitative predictions we use again the data for DOPC according to which the monolayer spontaneous curvature can be estimated as $J_S^0 = -0.1\ \text{nm}^{-1}$ (Zimmerberg and Kozlov, 2006). Based on these data, we estimate for the background value of the bilayer modulus of Gaussian curvature

$$\bar{\kappa}_B^0 = 2\bar{\kappa}_0 - 4\kappa_m J_S^0\delta \approx 0. \tag{9}$$

Note that a real value of $\bar{\kappa}_B^0$ may be slightly negative or positive depending on the exact membrane composition but for the sake of our semiquantitative analysis we will take it to vanish based on the above estimation. A more exact approach should use $\bar{\kappa}_B^0$ as a free parameter whose value could be found from comparison of the model prediction with the experimental results.

The insertion dependent part to $\bar{\kappa}_B$ consists of two contributions. One, denoted by, $\Delta\bar{\kappa}_{ins}^{(1)}$, comes from the insertion generated spontaneous curvature of the outer monolayer,

$$\Delta\bar{\kappa}_{ins}^{(1)} = -2\kappa_m^{out}J_S^{out}\delta. \tag{10}$$

The second, $\Delta\bar{\kappa}_{ins}^{(2)}$, is the insertion contribution to the outer monolayer modulus of Gaussian curvature $\bar{\kappa}_m^{out}$. Whereas the first contribution, $\Delta\bar{\kappa}_{ins}^{(1)}$, can be determined based on the previous computational results for the insertion generated spontaneous curvature of

the outer monolayer J_S^{out} (Campelo et al., 2008), the second contribution $\Delta\bar{\kappa}_{ins}^{(2)}$ has not been analyzed earlier and had to be computed. We performed this computation and compared $\Delta\bar{\kappa}_{ins}^{(1)}$ and $\Delta\bar{\kappa}_{ins}^{(2)}$ based on the possibility to present $-\kappa_m^{out}J_S^{out}$ and $\bar{\kappa}_m^{out}$ as a first and second moments of the trans-monolayer stress profile generated by the insertion in the outer monolayer (see e.g., Helfrich, 1990; Kozlov et al., 1989). We used the method of computation developed in Campelo et al. (2008). According to this analysis (data not showed), $\Delta\bar{\kappa}_{ins}^{(2)}$ is positive but its absolute value constitutes only about 10% of that of $\Delta\bar{\kappa}_{ins}^{(1)}$ so that on a semiquantitative level $\Delta\bar{\kappa}_{ins}^{(2)}$ can be neglected.

Based on the above considerations, the bilayer modulus of the Gaussian curvature is determined by the insertion generated spontaneous curvature of the outer monolayer according to

$$\bar{\kappa}_B = -2\kappa_m^{out}J_S^{out}\delta. \tag{11}$$

For small area fractions of insertions, $\phi << 1$, this expression can be simplified to

$$\bar{\kappa}_B = -2\kappa_m\delta\,\zeta_{inc}\phi, \tag{12}$$

where ζ_{inc} is an effective spontaneous curvature of one inclusion (Campelo et al., 2008). The value of ζ_{inc} varies between $\zeta_{inc} \approx 0.75$ nm^{-1} and $\zeta_{inc} \approx 0.5$ nm^{-1} depending on whether or not the monolayers are laterally coupled (Campelo et al., 2008). We will use a middle value of $\zeta_{inc} \approx 0.6$ nm^{-1}. As the energy of the Gaussian curvature changes only at the cylinder-to-vesicle transition, to simplify the common tangent calculations by keeping a semiquantitative accuracy of the predictions, we took the $\bar{\kappa}_B$ as a constant corresponding to the insertion area fraction of $\phi \approx 0.03$ which represents an average value between $\phi = 0$ describing the situation where membrane curvature is generated by pure scaffolds and $\phi \approx 0.07$ where the relevant curvatures of about $1/20$ nm are created solely by insertion of amphipathic helices (Campelo et al., 2008). Inserting into (12) the values of $\zeta_{inc} \approx 0.6$ nm^{-1} and $\phi \approx 0.03$ along with $\kappa_m = 4\ 10^{-20}$ Joule for the monolayer bending rigidity and $\delta = 1.5$ nm for the monolayer thickness, we obtain for the value of the bilayer modulus of Gaussian curvature of $\bar{\kappa}_B = -2\ \ 10^{-21}$ Joule, which was used in the computations. It has to be stressed again that using an average value of $\bar{\kappa}_B = -2\ \ 10^{-21}$ Joule pretends on a semiquantitative rather than quantitative character of the model predictions. Specifically, at large values of ϕ, where the values of the Gaussian modulus can be considerably more negative than the used number, membrane fission will be even more favorable than that predicted by the phase diagrams below.

Scaffold-free Energy

We describe the shape of a crescent like scaffold by the curvature of the line of its attachment to the membrane. We suppose that the intrinsic shape of a BAR domain corresponds to a circular arc so that the attachment line of the scaffold to the membrane is characterized by curvature C_p referred below to as the protein intrinsic curvature. Deformation of the BAR domain with respect to its intrinsic shape requires energy. We assume that this energy related to the unit length of the BAR-lipid attachment line is quadratic in the deviation of the line curvature C from its intrinsic value, $\varepsilon = 1/2\ \zeta_p(C - C_p)^2$ (Iglic et al., 2007), where ζ_p is the protein bending rigidity.

The bending energy of a whole BAR scaffold is obtained by integrating ε over the length of the attachment line. For the following, it will be convenient to characterize the scaffold by an elastic modulus having the same units as the membrane bending rigidity. We denote by w and L the effective width and length of the scaffold projection on the membrane plane, and define the BAR scaffold bending modulus by $\kappa_p = \zeta_p/w$. Using the relation, $A_p = wL$, we can express the scaffold energy per unit length as:

$$\varepsilon = \frac{1}{2}\kappa_p\frac{A_p}{L}(C - C_p)^2. \tag{13}$$

Denoting by N_p the number of the scaffolds in the system and by A_{tot} the total membrane area, the overall scaffold energy per unit area of membrane is:

$$f_p = \frac{N_p}{A_{tot}}\int_0^L \varepsilon(l)dl, \tag{14}$$

where the integration is performed over the length of the attachment line, dl, taking into account that, generally, the scaffold curvature depends on the position along the attachment line, $C(l)$.

It has to be noted that the only physical reason for a scaffold to deviate from its intrinsic curvature C_p and accumulate the elastic energy is its interaction with the membrane which is limited by the strength of attraction between the positively charged residues of the protein and the negatively charged lipid polar heads. Therefore, the protein elastic characteristics C_p and κ_p have an effective character reflecting interplay between the elastic stresses in the protein per se and the protein-lipid interaction. Here we assume for simplicity that the protein intrinsic curvature C_p is determined by the scaffold structure whereas the effects of the protein-membrane attachment strength are taken into account by the effective value of the protein rigidity, κ_p, which is considered, therefore, as a free parameter.

Translational Entropy of Proteins

To compute the translational entropy of the proteins on the membrane surface we represent the membrane as a grid with elementary cell area equal to that of a lipid molecule, A_l. The amount of the elementary cells covered by one protein is $a = A_p/A_l$. An approximate expression for the translational entropy of N_p proteins on a grid consisting of N elementary cells, which gives the correct entropy values for the limiting cases of low and full grid coverage, is $S = -k_B\, N/a[N_p a/N \ln(N_p a/N) + (1 - N_p a/N)\ln(1 - N_p a/N)]$. Taking into account that the total membrane area is $A_{tot} = A_l N_l + A_{inc} N_p$, the number of the elementary cells in the grid is $N = A_{tot}/A_l$, and the entropy contribution to the free energy related to the membrane unit area is $f_{ent} = -TS/A_{tot}$, we obtain using Equation 1,

$$f_{ent} = \frac{\kappa_B T}{A_p}\left[\frac{2x\,A_p}{A_l + xA_{ins}}\ln\left(\frac{2x\,A_p}{A_l + xA_{ins}}\right) + \left(1 - \frac{2x\,A_p}{A_l + xA_{ins}}\right)\ln\left(1 - \frac{2x\,A_p}{A_l + xA_{ins}}\right)\right]. \tag{15}$$

Energy of Protein Steric Interaction

To account for the states where the proteins cover, practically, the whole membrane surface, we introduce the energy of the steric repulsion between the proteins in the form

$$f_{ster} = \begin{cases} 0 & \text{if } \phi \le \tilde{\phi} \\ \dfrac{2x}{A_l + xA_{ins}} \cdot \Gamma\left(\phi - \tilde{\phi}\right)^2 & \text{if } \phi \ge \tilde{\phi} \end{cases}, \tag{16}$$

where $\tilde{\phi}$ is the insertion area fraction corresponding to the membrane coverage by proteins at which the latter start to repel each other sterically; Γ is the compression rigidity of the proteins whose large value limits, effectively, the amount of the proteins on the surface.

Energies of the Tubular and Vesicular Phases

The total energy per unit area of the membrane in each of the considered phases includes all the contributions above, $f = f_m + f_p + f_{ent} + f_{ster}$. For our analysis based on solution of Equations 2, we need the energy of each phase related to one lipid molecule, $g = A_{tot} \cdot f/N_l$.

In the tubular phase, the total curvature of the membrane J is related to the cylinder radius R by $J = 1/R$, whereas the Gaussian curvature equals zero, $K = 0$. Taking into account Equations 3, 4, 7, and 8, we obtain

$$g^c(x) = (A_l + xA_{ins})\left\{\frac{1}{2}\kappa^b(\phi)\left[\frac{1}{R(x)} - J_s^b(\phi)\right]^2 + f_{ent}(\phi) + f_{ster}(\phi)\right\} + \frac{1}{2}x \cdot \frac{\kappa_p A_p}{L}\int_0^L (C(l) - C_p)^2 dl \tag{17}$$

where the Equations 3, 5, 6, 15, and 16 have to be substituted for the membrane bending modulus $\kappa(\phi)$, spontaneous curvature $J_s^b(\phi)$, the contribution of the translational entropy $f_{ent}(\phi)$ and the steric interaction $f_{ster}(\phi)$. The scaffold curvature depends, according to standard geometrical relationships, on the orientation of the protein-membrane attachment line with respect to the cylinder axis and is inversely proportional to the cylinder radius $R(x)$. Our analysis shows that in the relevant range of the protein-to-lipid ratio, x, the average orientation of the scaffolds is perpendicular to the cylinder axis (see Appendix). This scaffold orientation will be considered below. To obtain the final form for the energy of the tubular phase, the cylinder radius $R(x)$ minimizing the energy (Equation 17) has to be found and inserted into the Equation 17. Because of the complex dependence of the last term in Equation 17 on the radius $R(x)$, we perform this latter step numerically. We neglect in Equation 17 the energy of the tubule end-cap assuming the tubules to be sufficiently long compared to their radii.

In the vesicular phase, the total membrane curvature is related to the sphere radius by $J = 2/R$, and the Gaussian curvature is $K = 1/R^2$. Due to the isotropy of the spherical surface, the curvature C of the scaffold-protein attachment lines does not depend on the scaffold orientation and is related to the sphere radius by $C = 1/R$. As a result, the energy of the vesicular phase related to one lipid molecule is given by

$$g^s(x) = (A_l + xA_{ins})\left\{\frac{1}{2}\kappa^b(\phi)\left[\frac{2}{R(\phi)} - J_s^b(\phi)\right]^2 + \frac{\bar{\kappa}}{R(x)^2} + f_{ent}(\phi) + f_{ster}(\phi)\right\} + \frac{1}{2}\cdot x \cdot \kappa_p \cdot A_p \cdot \left(\frac{1}{R} - C_p\right)^2, \tag{18}$$

where the vesicle radius minimizing the energy (Equation 18) and given by $1/R = 2\cdot(A_l + xA_{inc})\kappa(\phi)J_s^b(\phi) + x\cdot\kappa_p\cdot A_p\cdot C_p/4\cdot(A_l + xA_{inc})\kappa(\phi) + 2\cdot(A_l + xA_{inc})\cdot\bar{\kappa} + x\cdot\kappa_p\cdot A_p$, has to be substituted.

For illustration of the analysis, we present in Figure S1 (D-F (upper panels)) the energies of the vesicular and tubular phases as functions of the protein-to-lipid ratio x for specific values of the effective protein rigidity $\kappa_p = 410^{-19}$ Joule and of the monolayer modulus of Gaussian curvature $\bar{\kappa} = -2.10^{-21}$ Joule. The common tangent lines reveal the critical ratios x^{c*} and x^{s*}. The figures describe the cases of amphiphysin (A_{ins} = 12 nm^2) (Figure S1D), endophilin WT (A_{ins} = 20 nm^2) (Figure S1E) and endophilin DAH (A_{ins} = 30 nm^2) (Figure S1F). These figures show that, whereas for amphiphysin and endophilin WT both the tubular and vesicular phases can form and coexist, for endophilin DAH only the vesicular phase emerges from the initial flat membrane whereas the tubular phase is not expected at any protein-to-lipid ratio. The results analogous to those for endophilin DAH were also obtained for epsin

ENTH domains (not shown). In contrast for endophilin K8 which, practically, lacks the hydrophobic insertions, we obtained generation of the tubular phase in the absence of the vesicular one (not shown).

Model Predictions

We present the results of the theoretical analysis in the form of phase diagrams (Figures S1D–S1F, lower panels) showing the ranges of protein-to-lipid ratio x and of the ratio between the scaffold and the lipid monolayer bending moduli, κ_p/κ_m, corresponding to formation of the vesicular phase, tubular phase and coexistence between them. The computed phase diagrams presented here are for an N-BAR module comprising two N-terminal amphipathic helices (such as amphiphysin) (Figure S1D); an N-BAR module with an extra amphipathic domain in the middle of the domain in addition to the two N-terminal amphipathic helices (such as endophilin WT) (Figure S1E); an N-BAR domain having two doubled N-terminal amphipathic helices and the middle domain (such as endophilin-DAH mutant; see below and Figure S1F). The points corresponding to vanishing scaffold rigidity, $\kappa_p = 0$ (e.g., Figure S1D), describe the phase generated by a protein possessing a single amphipathic helix and producing no scaffolding effect (such as epsin ENTH domains). An extended phase diagram (Figure S1G) accounts for possible variations of the membrane modulus of Gaussian curvature $\bar{\kappa}_B$.

The major conclusion illustrated by all the phase diagrams (Figures S1D–S1F) is that shallow hydrophobic insertions favor membrane fission (on top of curvature generation) and hence support the vesicular phase. The crescent-like scaffolds support membrane bending without fission and, thus, favor the tubular phase counteracting the fission process. Indeed, all the phase diagrams (Figures S1D–S1F) predict the vesicular phase to form for protein rigidities tending to zero, $\kappa_p \rightarrow 0$, at which the scaffolding has a weak or no effect. If the scaffold rigidity κ_p exceeds a certain threshold value, κ_p^*, the tubular phase is predicted to be generated by amphiphysin (Figure S1D) and endophilin WT (Figure S1E) in a limited range of compositions x. The tubular phase either includes the whole system material (low x), or shares lipids and proteins with the vesicular phase (regions of phase coexistence at intermediate x). At relatively large protein-to-lipid ratios x, which may be irrelevant for real biological situations, the vesicular phase always prevails. Coexistence of vesicles and tubules is predicted to be observed for both amphiphysin and endophilin in a certain range of x. According to Figures S1D and S1E, the threshold scaffold rigidity κ_p^* is predicted to be larger for endophilin WT having two N-terminal insertions and the middle insertion domain than for amphiphysin possessing only the two N-terminal insertions. Finally, scaffolds with little or no insertions are predicted to generate tubulation but no fission (not shown at the phase diagrams).

It has to be emphasized again that the presented phase diagrams have a semiquantitative character and underestimate the extent of membrane fission and high insertion concentrations in the outer membrane monolayer. Moreover, the essence of the described effect of hydrophobic insertions on the membrane elastic properties relevant for fission is generation of negative values of the modulus of Gaussian curvature $\bar{\kappa}_B$ of the neck membrane. The dependence of membrane configurations on $\bar{\kappa}_B$ was predicted and extensively analyzed previously (see e.g. Huse and Leibler, 1991; Schwarz and Gompper, 1999).

It should be noted that the tubules observed to form upon protein action can represent either thermodynamically equilibrium structures predicted by our theoretical model or have a nonequilibrium transient character and transform into vesicles after a sufficiently long period of time. Our equilibrium phase diagrams do not account for the latter type of tubules. Specifically, our model predicts that shaping of the initial liposomes by proteins with solely or predominantly hydrophobic insertions, such as epsin ENTH or endophilin-DAH, should result (after equilibration) in vesiculation with no tubule formation. At the same time, in in vitro experiments with these proteins we observe a few tubules (of narrow diameter) in addition to many vesicles. These residual tubules could be transient nonequilibrium intermediates of the vesicle formation. In contrast, our calculations suggest that membrane shaping by a combined action of insertions and crescent-like scaffolds provided, for example, by N-BAR domains of endophilin and amphiphysin, can result in equilibrium coexistence between tubules and vesicles of similar curvatures. Indeed, tubules induced by these N-BAR domains are frequently seen together with the vesicles in vitro and in vivo, and the vesicle size tends toward the tubule diameter.

Appendix: Tilted BARs on Cylindrical Vesicles

The curvature of the BAR scaffolds is equal to the normal membrane curvature measured along the protein-membrane attachment line. When the membrane is a cylinder of radius R, the curvature of the scaffold at its center depends on the angle $\boldsymbol{\psi}$ between the direction of the scaffold and the cylinder axis (Figure S1H) and can adopt values between 0 and R^{-1}. The orientation that the BAR domains attain in the equilibrium state of the system depends on the relation between the spontaneous curvature of the scaffold and the spontaneous curvature of the membrane.

Consider first short scaffolds ($l << {C_p}^{-1}$) for which the curvature equals $C(\boldsymbol{\psi}) = sin^2(\psi)/R$. In case that $C_p < J_s$, both the scaffolds and the membrane in the equilibrium state are stress-free because they can, simultaneously, attain their spontaneous curvatures. Mathematically, this requirement is satisfied by solving the set of equations: $R = 1/J_S$ and $sin^2(\psi)/R = C_p$. Because $C_p < J_s^b$, a solution of this equation exists for $\boldsymbol{\psi} < \pi/2$. If $C_p > J_s$, the elastic energy of the system is minimal when the orientation of the scaffolds is perpendicular to the cylinder axis $\boldsymbol{\psi} = \pi/2$, because a tilted orientation would only decrease the curvature of the scaffolds and increase the curvature of the membrane and therefore will increase the elastic energy. Consequently, we propose that in the general case of scaffolds that are not necessarily short, the orientation of the scaffolds will satisfy:

$$\begin{cases} \psi \leqq \dfrac{\pi}{2} & C_p \leqq J_s^b \\ \psi = \dfrac{\pi}{2} & C_p \geqq J_s^b \end{cases} . \quad \text{(A1)}$$

The spontaneous curvature of the membrane J_s^b depends on the concentration of the insertions in the membrane (Campelo et al., 2008); it is small at low concentrations and increases monotonically with the increasing amount of the insertions in the system. Therefore, we expect that at low concentrations of N-BARs for which $C_p > J_s^b$ the orientation of the scaffolds will be perpendicular to the cylinder axis and for high concentrations where $C_p < J_s^b$ the scaffolds will adopt a tilted orientation with respect to the cylinder axis.

When the relation between the bending rigidities of the scaffolds and the lipid monolayer is unknown, there is a variety of shapes that the membrane and the scaffolds may form and, therefore, the equilibrium state of the system cannot be found analytically. However, if the scaffolds are very rigid relative to the membrane, $\kappa_p >> \kappa$, it is reasonable to assume that the shape of the scaffolds is round because in this case the curvature at any point along the scaffold should be close to its spontaneous curvature C_p. The membrane shape will depend on the scaffold orientation relative to the cylinder axis and, generally, will belong to the family of cylinders with elliptic cross-sections. If the scaffolds are very soft relative to the membrane, $\kappa_p \ll \kappa$, we expect that the shape of the membrane will be a circular cylinder with a curvature close to the spontaneous curvature of the membrane J_s^b, whereas the shape of a scaffold with a general orientation relative to the cylinder axis will be a part of an ellipse. We assume in this section that the scaffolds are all parallel to each other, as our calculations show that their orientation in equilibrium is narrowly distributed around an average value.

In the case of rigid scaffolds, the membrane cross-section is an ellipse with axes $a = R \cdot sin(\psi)$, $b = R$. We choose the following parameterization for the normal cross-section of the membrane:

$$\begin{cases} x = a \cdot \sin(t) \\ y = b \cdot \cos(t) \end{cases}, 0<t<2\pi \tag{A2}$$

and use the Equations 3, 4, 13, and 14. The free energy per lipid in this case is:

$$g^I(x,\psi) = (A_l + x\,A_{inc}) \left\{ \frac{1}{2}\kappa^b(\phi) \frac{\int_0^{2\pi} \left[\frac{sin(\psi)}{R(x,\psi)(cos^2(t)\,sin^2(\psi) + sin^2(t))^{\frac{3}{2}}} - J_s^b(\phi) \right]^2 \sqrt{cos^2(t)\,sin^2(\psi) + sin^2(t)}dt}{\int_0^{2\pi} \sqrt{cos^2(t)\,sin^2(\psi) + sin^2(t)}} + f_{ent}(\phi) + f_{ster}(\phi) \right\} + \frac{1}{2} \cdot x \cdot \kappa_p \cdot A_p \cdot \left(\frac{1}{R} - C_s \right)^2 \tag{A3}$$

Minimization of the energy (Equation A3) with respect to the radius of the scaffolds R and the tilt angle ψ gives the equilibrium state of the system. The results of the numerical calculation of the tilt angle as a function of the protein-to-lipid ratio x for rigid scaffolds of endophilin, $\kappa_p/\kappa = 1000$, is shown in Figure S1I. The kink in the curve corresponds to the critical protein-to-lipid ratio $x^{tilt} \approx 5.3 \cdot 10^{-3}$ for which the orientation of the scaffolds on the membrane starts deviating from $\pi/2$. The proposed criterion for tilting $J_s\,(x^{tilt}) = C_p$ is satisfied in this case.

Next we address the question whether we should ever observe the scaffold tilting with respect to the cylinder axis ($\psi < \pi/2$). Recall that according to the results of our analysis, the tubular phase is stable only for low protein-to-lipid ratios $x < x^{*c}$. According to our calculations, for both endophilin WT and amphiphysin N-BAR domains the relationship between the critical molar ratios is $x^{*c} < x^{tilt}$. This means that in the whole range of stability of the tubular phase $x < x^{tilt}$ the scaffold orientation is perpendicular to the cylinder axis. The system compositions at which the scaffolds tend to adopt a tilted orientation with respect to the cylinder axis lie outside of the range of stability of the tubular phase. Hence the possibility of the scaffold tilting does not change the phase diagrams for the N-BAR-lipid systems discussed in the main text.

In the case of rigid membrane, $\kappa_p \ll \kappa$, the membrane shape is a cylinder of radius $R\,(x, \psi)$ and the contour of the scaffolds is a part of an ellipse with axes $a = R/sin(\psi)$ and $b = R$. Choosing the same parameterization as above (Equation A2) and using the Equations 3, 4, 7, and 8, we obtain for the free energy per lipid:

$$g^{II}(x,\psi) = (A_l + x\,A_{inc}) \left\{ \frac{1}{2}\kappa^b(\phi) \left[\frac{1}{R(\phi)} - J_s^b(\phi) \right]^2 + f_{ent}(\phi) + f_{ster}(\phi) \right\} + \frac{1}{2} \cdot x \cdot \kappa_p \cdot A_p \tag{A4}$$

where the limits of integration have to be found numerically by solving:

$$l = \int_{-\varphi}^{\varphi} \sqrt{\frac{R^2}{sin^2(\psi)} cos^2(t) + R^2 sin^2(t)}dt. \tag{A5}$$

Repeating the same procedure as in the case of rigid scaffolds we find again that at low protein-to-lipid ratios $x < x^{tilt}$ the free energy of the system is minimized at $\psi = \pi/2$ and at high concentrations of N-BARs, $x > x^{tilt}$, the scaffolds attain a tilted orientation. The critical protein-to-lipid ratio x^{tilt} is the same as in the previous case.

In all the cases we studied we obtained $x^{tilt} > x^{*c}$. As explained above, this means that in all systems that undergo a phase transition from the tubular phase to the vesicular phase the orientation of the scaffolds in the tubular phase is perpendicular to the cylinder axis.

We conclude this section by an analytic derivation of the criterion for tilting for the case of long scaffolds that are very rigid relative to the membrane, and therefore are not deformed and attain their spontaneous curvature. As the scaffolds are extremely rigid, $R = 1/C_p$, the system energy is obtained by substituting $R = 1/C_p$ into the energy g^I (Equation A3). The energy depends on the orientation of the scaffolds ψ:

$$f(x,\psi) \equiv \frac{\int_0^{2\pi} \left[C_p \frac{\sin(\psi)}{(\cos^2(t)\sin^2(\psi) + \sin^2(t))^{\frac{3}{2}}} - J_s(\phi) \right]^2 \sqrt{\cos^2(t)\sin^2(\psi) + \sin^2(t)}dt}{\int_0^{2\pi} \sqrt{\cos^2(t)\sin^2(\psi) + \sin^2(t)}} \tag{A6}$$

We investigate the orientation of the scaffolds in equilibrium by evaluating the energy g^I in the proximity of $\psi = \pi/2$. To this end we write: $f(\psi) = f(\pi/2) + (\psi - \pi/2)f'(\pi/2) + 1/2(\psi - \pi/2)^2 f''(\pi/2)$. Now, note that $f'(\pi/2) = 0$ and the sign of the second derivative $f''(\pi/2)$ depends on the relation between the spontaneous rigidities of the scaffold and the membrane:

$$\begin{cases} f''\left(\frac{\pi}{2}\right) \geqq 0 & \text{for } C_s \geqq J_s^b \\ f''\left(\frac{\pi}{2}\right) \leqq 0 & \text{for } C_s \leqq J_s^b \end{cases}. \tag{A7}$$

Substitution of this result in the energy Equation A6 proves again that the criterion for tilting for extremely rigid scaffolds is $J_s^b = C_p$.

SUPPLEMENTAL REFERENCES

Boulant, S., Kural, C., Zeeh, J.C., Ubelmann, F., and Kirchhausen, T. (2011). Actin dynamics counteract membrane tension during clathrin-mediated endocytosis. Nat. Cell Biol. *13*, 1124–1131.

Campelo, F., McMahon, H.T., and Kozlov, M.M. (2008). The hydrophobic insertion mechanism of membrane curvature generation by proteins. Biophys. J. *95*, 2325–2339.

Doyon, J.B., Zeitler, B., Cheng, J., Cheng, A.T., Cherone, J.M., Santiago, Y., Lee, A.H., Vo, T.D., Doyon, Y., Miller, J.C., et al. (2011). Rapid and efficient clathrin-mediated endocytosis revealed in genome-edited mammalian cells. Nat. Cell Biol. *13*, 331–337.

Ehrlich, M., Boll, W., Van Oijen, A., Hariharan, R., Chandran, K., Nibert, M.L., and Kirchhausen, T. (2004). Endocytosis by random initiation and stabilization of clathrin-coated pits. Cell *118*, 591–605.

Ezratty, E.J., Bertaux, C., Marcantonio, E.E., and Gundersen, G.G. (2009). Clathrin mediates integrin endocytosis for focal adhesion disassembly in migrating cells. J. Cell Biol. *187*, 733–747.

Ford, M.G., Mills, I.G., Peter, B.J., Vallis, Y., Praefcke, G.J., Evans, P.R., and McMahon, H.T. (2002). Curvature of clathrin-coated pits driven by epsin. Nature *419*, 361–366.

Gallop, J.L., Jao, C.C., Kent, H.M., Butler, P.J., Evans, P.R., Langen, R., and McMahon, H.T. (2006). Mechanism of endophilin N-BAR domain-mediated membrane curvature. EMBO J. *25*, 2898–2910.

Helfrich, W. (1973). Elastic properties of lipid bilayers: theory and possible experiments. Z. Naturforsch. C *28*, 693–703.

Helfrich, W. (1990). Elasticity and thermal undulations of fluid films of amphiphiles. In Les Houches, 1988 - Liquids and interfaces, J. Charvolin, J.-F. Joanny, and J. Zinn-Justin, eds., pp. 212–237.

Henne, W.M., Boucrot, E., Meinecke, M., Evergren, E., Vallis, Y., Mittal, R., and McMahon, H.T. (2010). FCHo proteins are nucleators of clathrin-mediated endocytosis. Science *328*, 1281–1284.

Hillert, M. (1998). Phase equilibria, phase diagrams, and phase transformations: their thermodynamic basis (Cambridge: Cambridge University Press).

Huse, D.A., and Leibler, S. (1991). Are sponge phases of membranes experimental gauge-Higgs systems? Phys. Rev. Lett. *66*, 437–440.

Iglic, A., Slivnik, T., and Kralj-Iglic, V. (2007). Elastic properties of biological membranes influenced by attached proteins. J. Biomech. *40*, 2492–2500.

Kozlov, M.M., and Helfrich, W. (1992). Effects of a Cosurfactant on the Stretching and Bending Elasticities of a Surfactant Monolayer. Langmuir *8*, 2792–2797.

Kozlov, M.M., Leikin, S.L., and Markin, V.S. (1989). Elastic properties of interfaces. Elasticity moduli and spontaneous geometrical characteristics. J. Chem. Soc., Faraday Trans. II *85*, 277–292.

Kozlovsky, Y., Efrat, A., Siegel, D.P., and Kozlov, M.M. (2004). Stalk phase formation: effects of dehydration and saddle splay modulus. Biophys. J. *87*, 2508–2521.

Macia, E., Ehrlich, M., Massol, R., Boucrot, E., Brunner, C., and Kirchhausen, T. (2006). Dynasore, a cell-permeable inhibitor of dynamin. Dev. Cell *10*, 839–850.

Motley, A., Bright, N.A., Seaman, M.N., and Robinson, M.S. (2003). Clathrin-mediated endocytosis in AP-2-depleted cells. J. Cell Biol. *162*, 909–918.

Niggemann, G., Kummrow, M., and Helfrich, W. (1995). The bending rigidity of phosphatidylcholine bilayers. Dependence on experimental methods, sample cell sealing and temperature. J. Phys. II *5*, 413–425.

Peter, B.J., Kent, H.M., Mills, I.G., Vallis, Y., Butler, P.J., Evans, P.R., and McMahon, H.T. (2004). BAR domains as sensors of membrane curvature: the amphiphysin BAR structure. Science *303*, 495–499.

Schwarz, U.S., and Gompper, G. (1999). Systematic approach to bicontinuous cubic phases in ternary amphiphilic systems. Phys. Rev. E Stat. Phys. Plasmas Fluids Relat. Interdiscip. Topics *59* (*5 Pt B*), 5528–5541.

Shaner, N.C., Lin, M.Z., McKeown, M.R., Steinbach, P.A., Hazelwood, K.L., Davidson, M.W., and Tsien, R.Y. (2008). Improving the photostability of bright monomeric orange and red fluorescent proteins. Nat. Methods *5*, 545–551.

Siegel, D.P., and Kozlov, M.M. (2004). The gaussian curvature elastic modulus of N-monomethylated dioleoylphosphatidylethanolamine: relevance to membrane fusion and lipid phase behavior. Biophys. J. *87*, 366–374.

Spivak, M. (1970). A Comprehensive Introduction to Differential Geometry (Waltham, MA: Brandeis University).

Templer, R.H., Khoo, B.J., and Seddon, J.M. (1998). Gaussian curvature modulus of an amphiphilic monolayer. Langmuir *14*, 7427–7434.

Wigge, P., Köhler, K., Vallis, Y., Doyle, C.A., Owen, D., Hunt, S.P., and McMahon, H.T. (1997). Amphiphysin heterodimers: potential role in clathrin-mediated endocytosis. Mol. Biol. Cell *8*, 2003–2015.

Zimmerberg, J., and Kozlov, M.M. (2006). How proteins produce cellular membrane curvature. Nat. Rev. Mol. Cell Biol. *7*, 9–19.

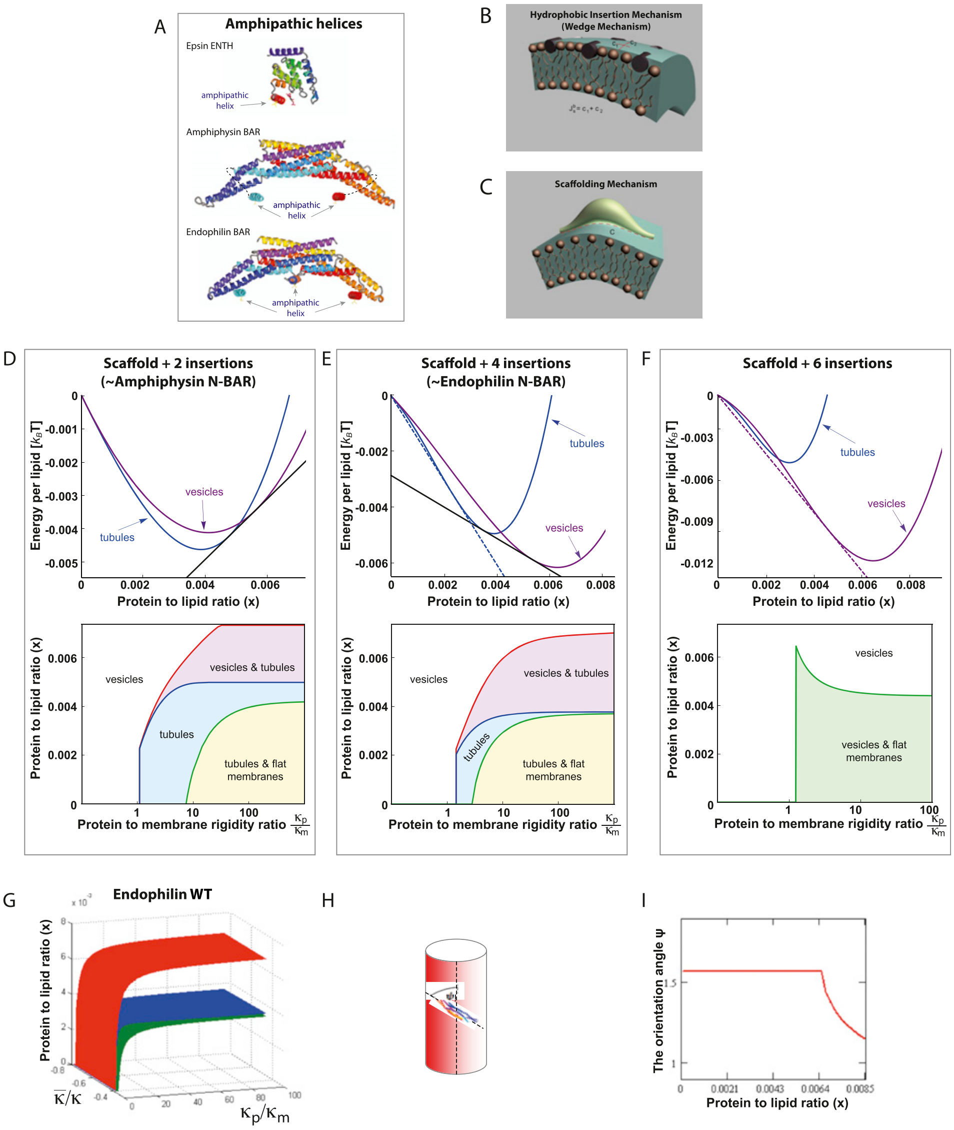

Figure S1. Predicted Membrane-Shaping Effects of Hydrophobic Insertions and Crescent-like Scaffolds, Related to Figure 1

(A) Structures of Epsin ENTH domain (PDB: 1h0a), Amphiphysin BAR domain (PDB: 1uru), and endophilin BAR domain (PDB: 2c08) (Ford et al., 2002; Gallop et al., 2006; Peter et al., 2004). Epsin ribbon diagram is colored from N-C in red to magenta. Amphiphysin and endophilin monomer 1 is colored N-C in red to yellow and monomer 2 is colored in cyan to magenta. The amphipathic helix of epsin 1 ENTH domain is folded around the head group of PtdIns(4,5)P_2 – Ins(3,4,5)P_3. For amphiphysin and endophilin the terminal amphipathic helices are not present in the structures and so are connected to the structures by dotted lines.

(B) Hydrophobic insertion mechanism.

(C) Scaffolding mechanism.

(D–F, upper panels) The free energies per lipid in the tubular (solid purple line) and vesicular (solid green line). The energies are plotted as a function of the protein-to-lipid ratio x for the effective protein rigidity $\kappa_p = 4 \cdot 10^{-19}$ Joule, and the monolayer modulus of Gaussian curvature $\bar{\kappa} = -\mathbf{2 \cdot 10^{-21}}$ Joule. The straight dashed lines indicate the common tangents to the energy curves determining the phase compositions at phase transitions. The in-plane area of hydrophobic insertions per one proteins scaffold A_{ins} is taken to be (D) A_{ins} = 12 nm^2 for amphiphysin. (E) A_{ins} = 20 nm^2 or endophilin WT. (F) A_{ins} = 30 nm^2 for endophilin DAH.

(D–F, lower panels). The phase diagrams showing the ranges of the protein-to-lipid ratio, x, and the ratios between the bending rigidities of a scaffold, κ_p, and a lipid monolayer,κ_m, for which the initially flat membranes undergo bending and fission (vesicular phase); bending without fission (tubular phase), or coexistence of the two regimes. The monolayer bending modulus and the modulus of Gaussian curvature are taken $\boldsymbol{\kappa = 4 \cdot 10^{-20}}$ Joule and $\bar{\kappa} = -\mathbf{2 \cdot 10^{-21}}$Joule, respectively, the effective spontaneous curvature of insertion is ζ_s = 0.75 nm^{-1}.

(G) Phase diagram taking onto account variations of the membrane modulus of Gaussian curvature $\bar{\kappa}_B$ for endophilin WT with the total insertion area per scaffold A_{ins} = 20 nm^2. Other parameters are as in (D)–(F).

(H) An illustration of the tilt angle of scaffolds on cylindrical vesicles. The tilt angle ψ is the angle that the elongated direction of the N-BAR scaffold *(colorful coils)* makes with the cylinder axis. The curvature of the scaffold at its center varies with the scaffold orientation, assuming values between R^{-1} and zero.

(I) The orientation of the scaffolds in the tubular phase relative to the cylinder axis ψ as a function of the protein-to-lipid ratio x for amphiphysin. For low N-BAR concentrations the scaffolds are perpendicular to the axis $\psi = \pi/2$. Above the critical ratio x $\approx$0.0128, the orientation angle ψ begins to deviate from $\pi/2$.

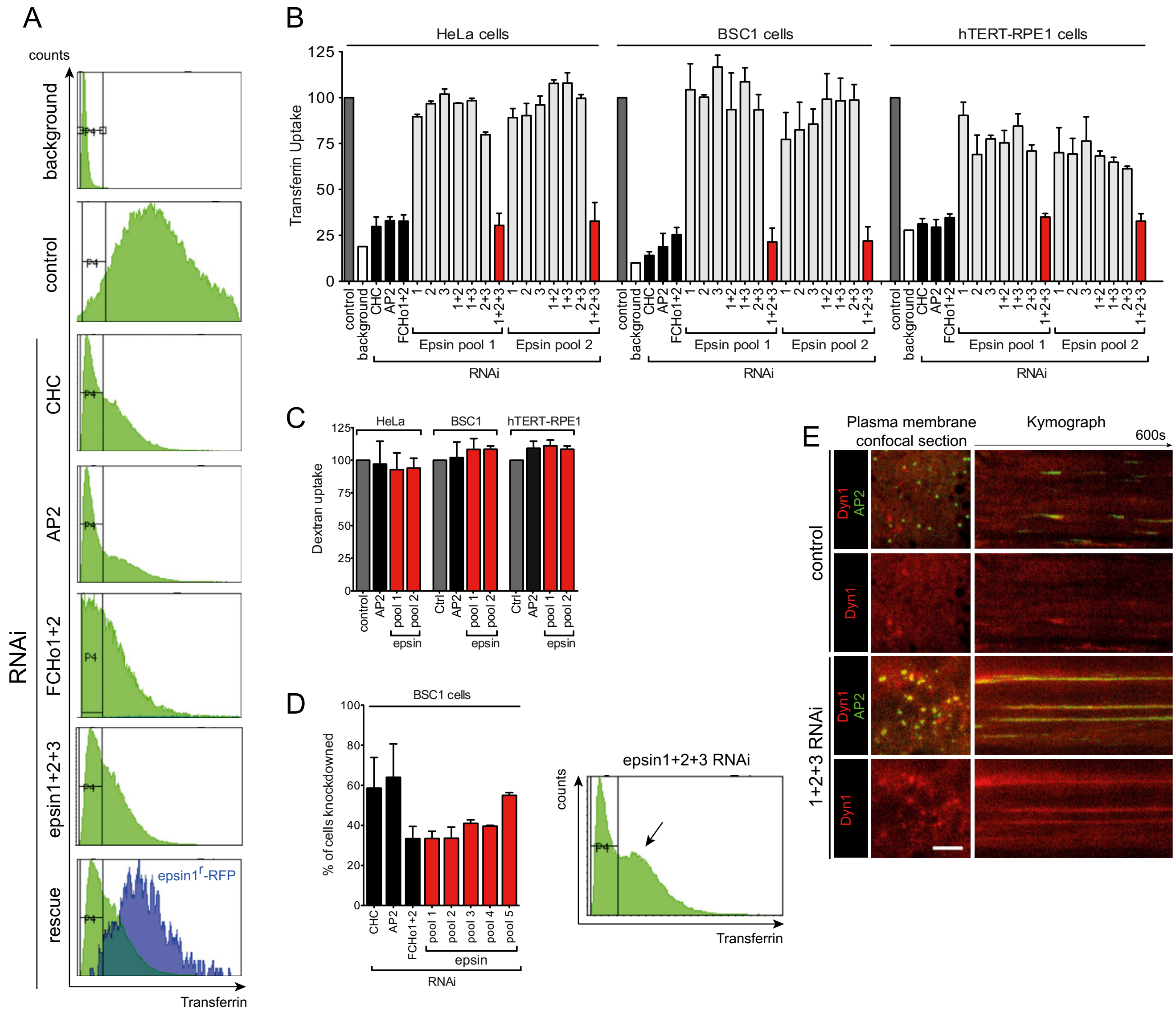

Figure S2. Epsin Is Required for CCV Scission, Related to Figure 2

(A) Representative FACS profiles of transferrin uptake (20 µg/ml for 7 min at 37°C) in BSC1 cells treated with control, clathrin (CHC), AP2, FCHO1+2, or Epsin1+2+3 pool 1 siRNA (72 hr prior to ligand uptake) or Epsin1+2+3 pool 1 rescued by expression of rat epsin1-RFP (blue).

(B) Effect of RNAi, using the siRNA pools 1 and 2, of epsin proteins on transferrin uptake (20 µg/ml for 7 min at 37°C) measured by flow cytometry. CHC, AP2, and FCHO protein depletions were used as positive controls (black bars). The values were normalized to the mean of the control cells (gray bars). The background (cells without transferrin) for each cell line is shown (white bars, background). Data are the mean ± SD.

(C) Effect of RNAi, using the siRNA pool1 and 2, of epsin proteins on dextran uptake (1 mg/ml for 15 min at 37°C) measured by flow cytometry. AP2 depletion was used as positive controls (black bars). The values were normalized to the mean of the control cells (gray bars). Data are the mean ± SD.

(D) Percentage of cells knocked down for the target proteins, as measured by the proportion of cells presenting a decrease in transferrin uptake down to background levels (‘P4’ region in A). Please note that less than half of the cells in FCHo1+2 and Epsin1+2+3 RNAi pools were strongly knocked down. Right, example of FACS profile of 2 populations (one strongly inhibited, ‘P4’ region, and another partially, ‘arrow’) in an Epsin1+2+3 RNAi sample. Data are the mean ± SD.

(E) Effect of epsin1+2+3 RNAi on recruitment of rat dynamin1 (red) and AP2 (σ2-EGFP, green). Bar, 5 µm.

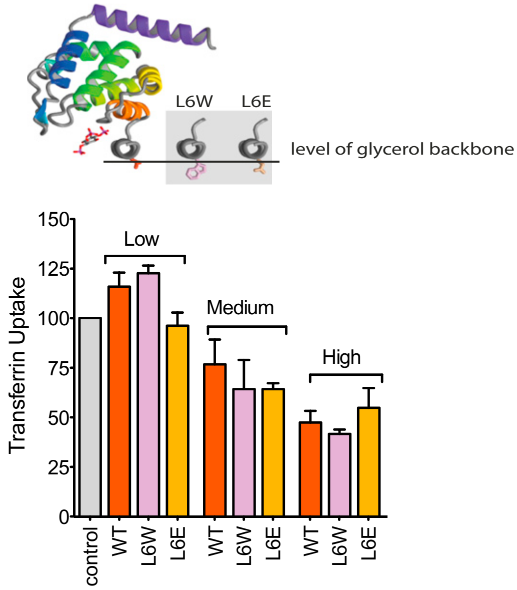

Figure S3. Epsin Can Mediate CCV Scission in Dynamin-Depleted Cells, Related to Figure 3

Top, scheme depicting the level of insertion of WT (leucine 6 in red) and L6W and L6E mutants. Bottom, effect of various Epsin1 WT, L6W, and L6E overexpression levels on transferrin uptake (20 μg/ml for 7 min at 37°C) measured by flow cytometry. "Low," "Medium,: and "High" corresponded to 10 ng, 50 ng, and 200 ng of epsin-encoding DNA per 2 × 10^4 cells, respectively. Data are the mean ± SD.

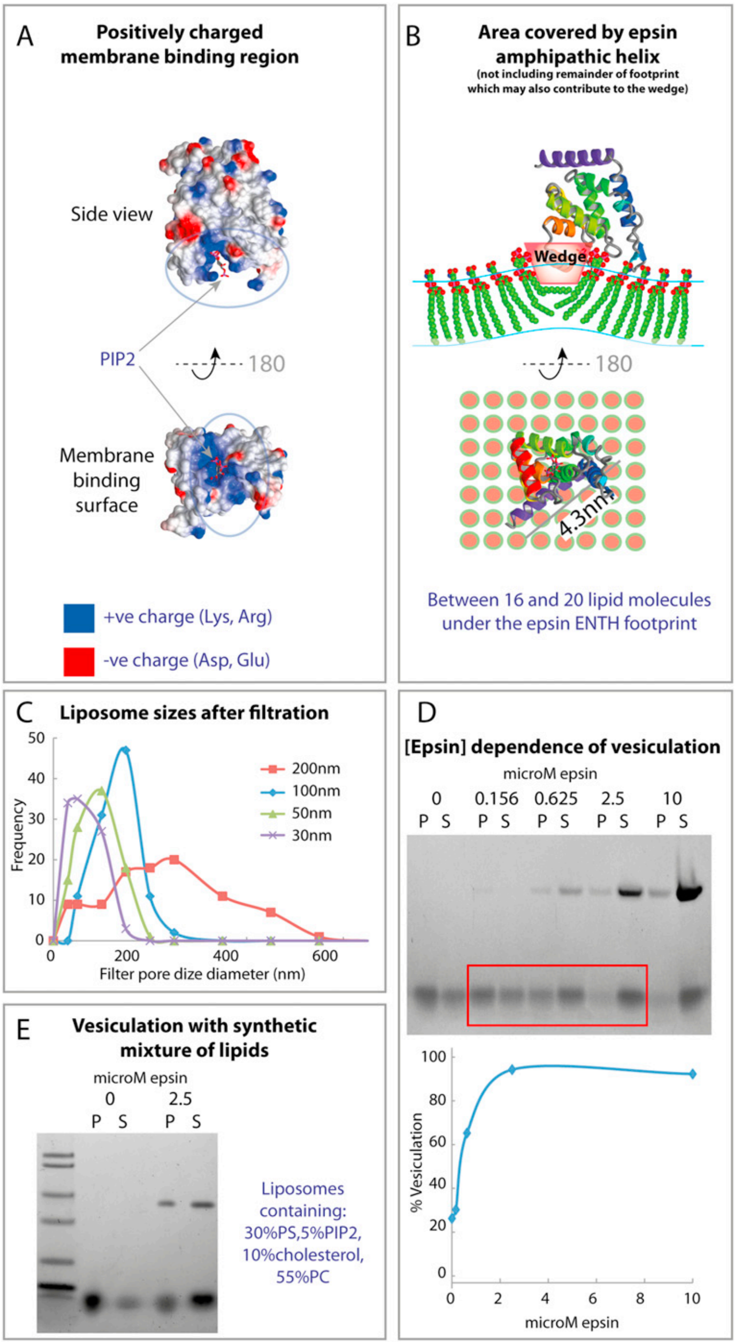

Figure S4. Epsin ENTH Domain Causes Extensive Membrane Vesiculation, Related to Figure 4

(A) Surface charge of epsin ENTH domain. A highly positively charged region surrounded by hydrophobic surface residues acts as the binding site for the head group of PtdIns(4,5)P_2 (PIP_2). All ribbon diagrams and surface representations are to scale.

(B) Model of how lipids must tilt and splay around the wedge-like shallow insertions of epsin amphipathic helix. Lipids are shown with red head groups and green acyl chains. A view directly down on the surface of the membrane gives a bird-eye view of the area covered by the ENTH domain.

(C) Liposome size distribution after filtration to various diameters indicated. 100 liposomes for each category were measured and binned to the sizes indicated by the symbols.

(D) Concentration dependence for epsin WT vesiculation of 0.125 mg/ml Folch liposomes at room temperature. Vesiculation is assessed as the percentage of lipid found in the supernatant after centrifugation.

(E) Vesiculation by of a synthetic mixture of lipids containing 5% PtdIns(4,5)P_2.

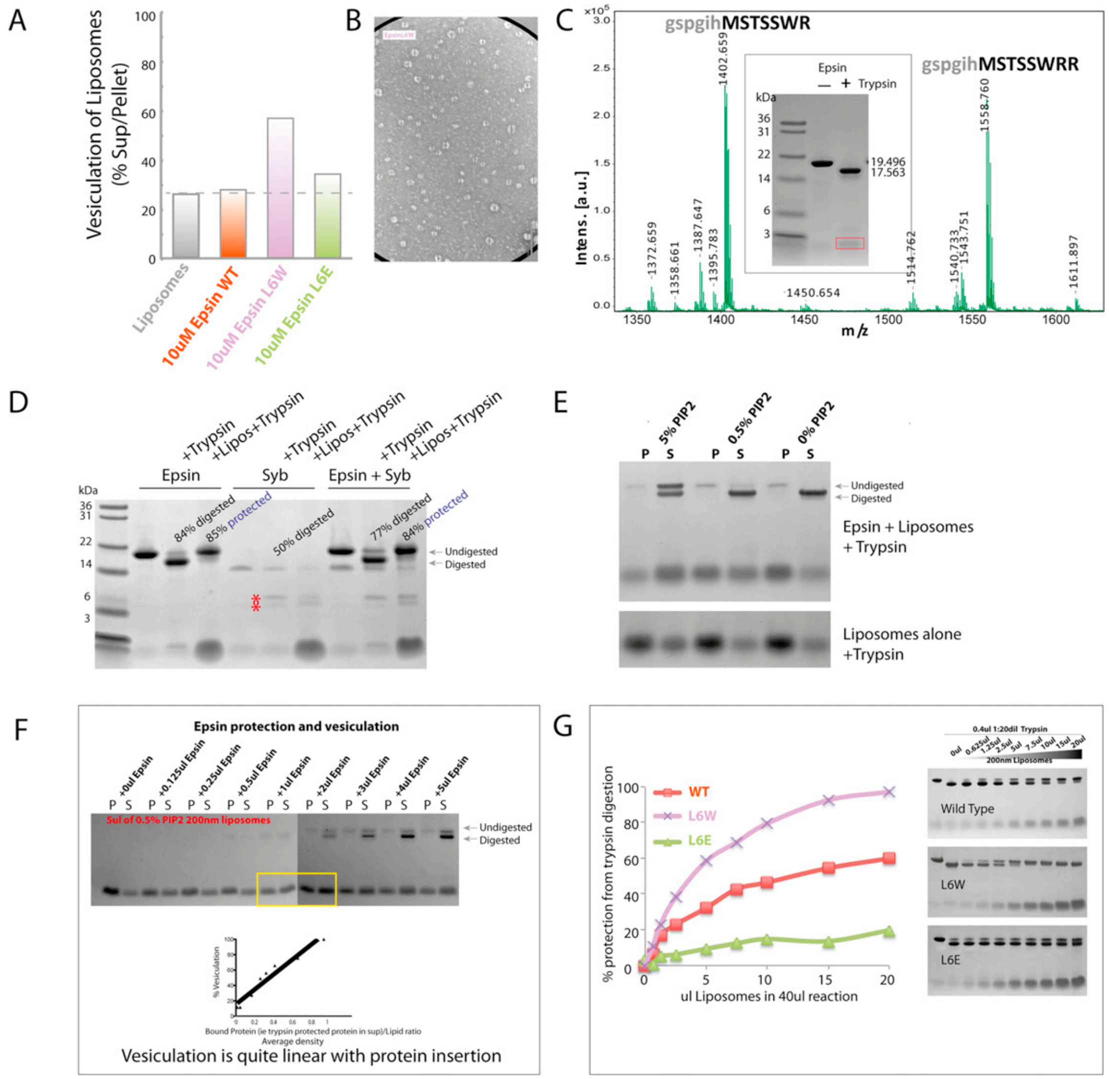

Figure S5. Membrane Vesiculation Is due to Amphipathic Helix Insertion, Related to Figure 5

(A) At 4°C epsin vesiculation is much less efficient. After 30 min incubation with 200 nm Folch liposomes only L6W is successful in giving a partial shift of liposomes from the pellet to the supernatant in the sedimentation assay.

(B) Representative EM of liposomes after epsin L6W, used to quantitate the diameter of small vesicles shown in (A). Smaller particles (micelles or protein aggregates) were not counted.

(C) Mass spectrometry of the complete trypsin digested sample of L6W epsin ENTH domain gave 2 prominent low molecular mass peptides (red box) whose sequence shows they are digested at 2 adjacent arginines. Arg8 coordinates the 4′ phosphate of the PIP2 inositol ring, whereas Arg7 coordinates the phosophodiester linkage (Ford et al., 2002). Removal of these residues by proteolysis means that the protein no longer binds membranes (Figure 2D). The mass of the proteolysed parent ENTH domain is consistent with a further cleavage of the amphipathic helix to the next lysine. This 3 amino acid peptide was not recovered.

(D) Trypsin itself is not inhibited by membranes. The possibility that trypsin is absorbed/inhibited by membranes was tested by taking an unfolded protein (synaptobrevin) which gives distinct cleavage products (*) and adding this to the liposome mixture. Proteins were preincubated with liposomes for 10 min before trypsin addition. Trypsin digestion of synaptobrevin is not inhibited by the addition of liposomes and may even be slightly enhanced.

(E) Epsin binding to synthetic liposomes (30% PS, 10% cholesterol, 55%–60% PC) with various PIP2 contents. Samples were subjected to 15 min incubation with trypsin to eliminate uninserted protein, showing that the limited amount of protected protein in the presence of 0.5% PIP2 was sufficient to give significant vesiculation.

(F) Increasing concentrations of epsin promote more extensive vesiculation of liposomes. After trypsin cleavage it becomes clear that, at higher concentrations, most of the added epsin is not bound/protected by the liposomes and so does not contribute to vesiculation. When vesiculation is plotted versus the bound/protected protein, vesiculation is linear relative to inserted epsin protein. The yellow box indicates the point of approximately 50% vesiculation where there is maximally 10% membrane coverage by epsin (assuming that 6ul epsin is giving saturation).

(G) Membrane binding of epsin ENTH domain and mutants. It is not easy to assess the amount of epsin bound to membrane by the traditional sedimentation assay as the protein causes a shift in the liposomes sedimentation pattern. However given that amphipathic helix insertion is the major event one wants to monitor and is a reflection of binding, we can use trypsin sensitivity of the protein as a measure of insertion. At room temperature for 30 min L6W mutant binds better than WT protein, which binds better than L6E mutant protein.

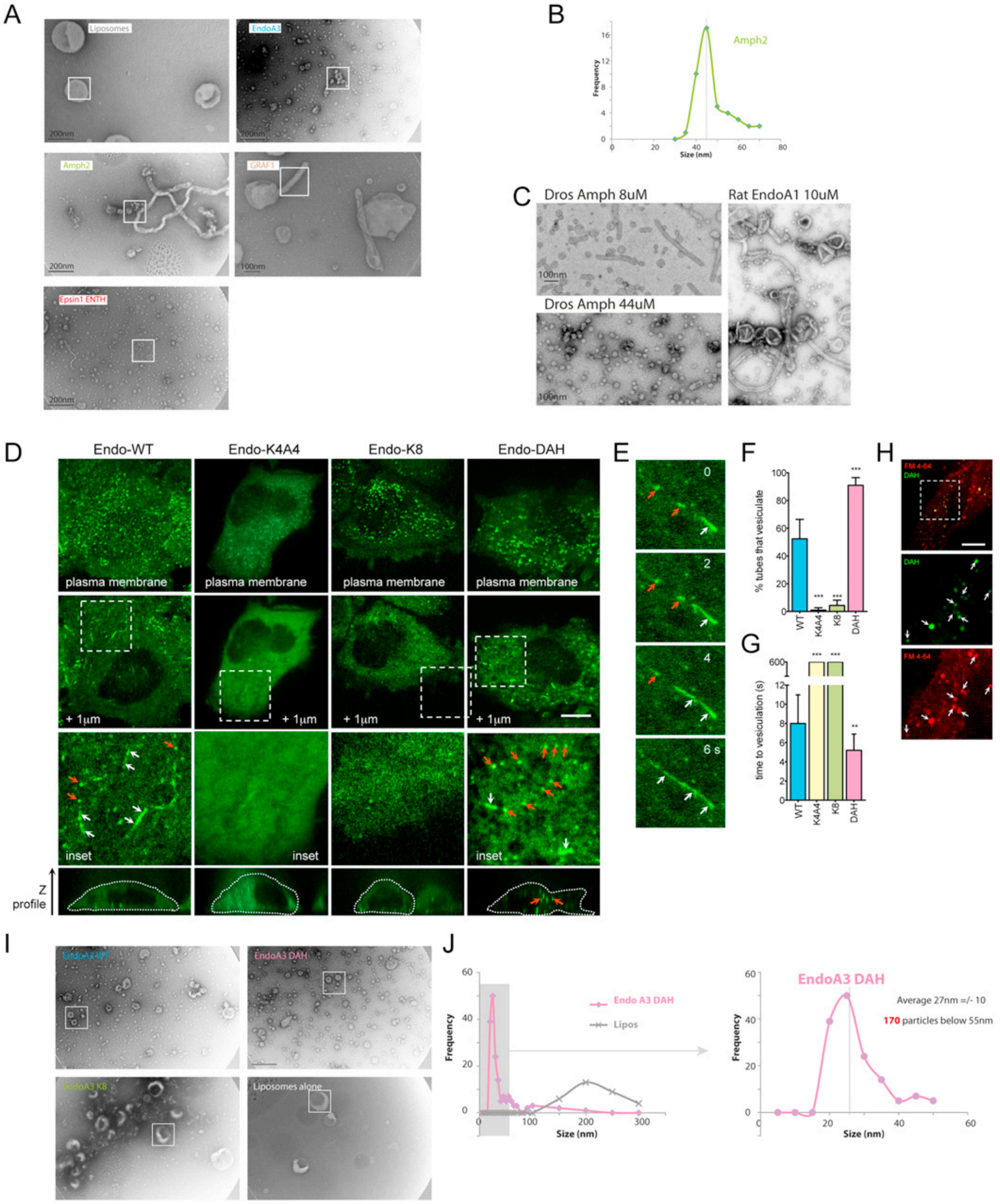

Figure S6. BAR Scaffolds Restrain Membrane Scission Catalyzed by Extensive Hydrophobic Insertions, Related to Figure 6

(A) Electron micrographs of liposomes treated with various BAR-domain proteins. White boxes are the areas detailed in the corresponding main figure.

(B) Size distribution of vesicles for amphiphysin2. Tubules and vesicles above 70 nm were excluded.

(C) Examples of vesiculation by BAR domains taken from previous figures (Gallop et al., 2006; Peter et al., 2004). It had previously been noted that at higher concentrations *D. melanogaster* amphiphysin BAR domain tended to produce vesicles of a rather uniform size.

(D) Confocal images of live HeLa cells expressing the respective endophilin constructs. First row represents the first focal plane at the bottom of the cells (“plasma membrane”). Note the presence of puncta with all four constructs. The second row represents a focal plane took 1 micron above the plasma membrane. The third row displays the inset of the boxed region. Note the tubules (white arrows) and the internal vesicles (red arrows). The last row shows a Z profile of each cell.

(E) Time-lapse imaging of vesicle formation. EndophilinA3 tubule (white arrows) vesiculating and forming vesicle (red arrows).

(F) Histogram reports the percentage of tubules that vesiculated for each constructs.

(G) Histogram depicts the time (average ± standard error of the mean [SEM]) to vesiculation of 50 tubules for each constructs (10 tubules for K4A4 constructs as they were rare). The tubules formed by the K4A4 and K8 constructs were stable and did not vesiculate during the time of imaging (600 s). Significance determined using Student’s t test (**p < 0.001).

(H) Electron micrographs of liposomes treated with various endophilin constructs (Endo-WT and mutants of the N-terminal amphipathic helix, DAH, double amphipathic helix and K8, N-terminal helix replaced by a stretch of 8 lysines). Liposomes were incubated for 60 min at 37°C with 2 μM protein. White boxes are the areas detailed in the corresponding main figure.

(I) Size distribution of vesicles for Endo-DAH (pink) and starting liposomes (gray).

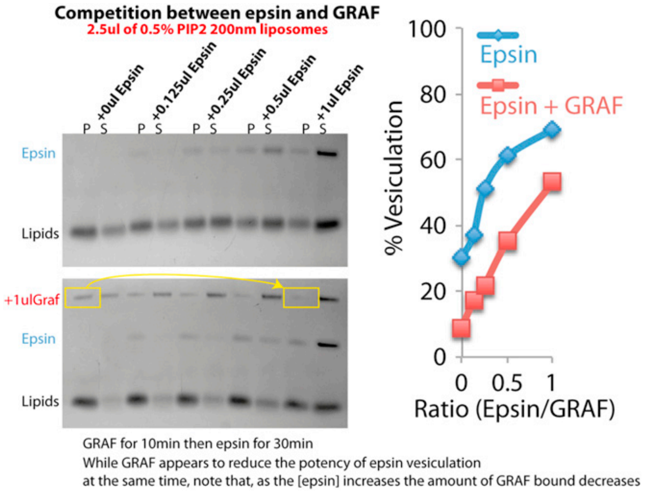

Figure S7. GRAF BAR+PH Domain Resists Epsin Vesiculation Activity, Related to Figure 7

Competition between epsin ENTH domain, which promotes vesiculation, and GRAF BAR+PH domain, which promotes membrane tubulation. Although GRAF does indeed appear to restrain the vesiculation, this effect can be accounted for by the reduced binding of epsin in the presence of GRAF.
